# Hydrocarbon Soluble Alkali‐Metal‐Aluminium Hydride Surrog[ATES]

**DOI:** 10.1002/chem.202201085

**Published:** 2022-08-11

**Authors:** Sumanta Banerjee, Peter A. Macdonald, Samantha A. Orr, Alan R. Kennedy, Alexander van Teijlingen, Stuart D. Robertson, Tell Tuttle, Robert E. Mulvey

**Affiliations:** ^1^ *West*CHEM, Department of Pure and Applied Chemistry University of Strathclyde Glasgow G1 1XL UK

**Keywords:** alkali metal aluminates, dihydropyridine, heterobimetallic complexes, hydride, London dispersion forces

## Abstract

A series of group 1 hydrocarbon‐soluble donor free aluminates [AM(^t^BuDHP)(TMP)Al(^i^Bu)_2_] (AM=Li, Na, K, Rb) have been synthesised by combining an alkali metal dihydropyridyl unit [(2‐^t^BuC_5_H_5_N)AM)] containing a surrogate hydride (sp^3^ C−H) with [(^i^Bu)_2_Al(TMP)]. These aluminates have been characterised by X‐ray crystallography and NMR spectroscopy. While the lithium aluminate forms a monomer, the heavier alkali metal aluminates exist as polymeric chains propagated by non‐covalent interactions between the alkali metal cations and the alkyldihydropyridyl units. Solvates [(THF)Li(^t^BuDHP)(TMP)Al(^i^Bu)_2_] and [(TMEDA)Na(^t^BuDHP)(TMP)Al(^i^Bu)_2_] have also been crystallographically characterised. Theoretical calculations show how the dispersion forces tend to increase on moving from Li to Rb, as opposed to the electrostatic forces of stabilization, which are orders of magnitude more significant. Having unique structural features, these bimetallic compounds can be considered as starting points for exploring unique reactivity trends as alkali‐metal‐aluminium hydride surrog[ATES].

## Graphical abstract

**Saturated 2,2,6,6‐tetramethylpiperidide**, TMP, has long played a key role in synthesis, here combined with an unsaturated dihydropyridine unit, 2‐*t*BuC_5_H_5_N, both contribute to the stability of a series of alkali metal aluminate structures by engaging in non‐covalent interactions with alkali metal cations, the extent of which depends on the alkali metal.

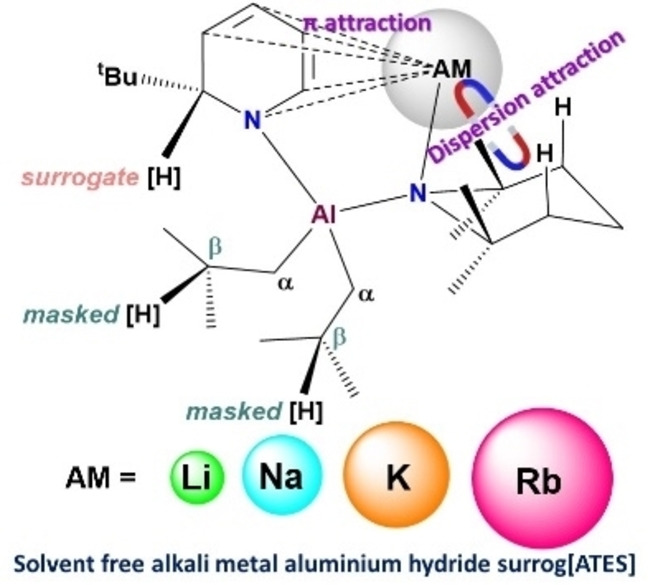

## Introduction

A recent seminal review highlighted the growing body of knowledge on molecular main group metal hydrides.^[1]^ These compounds can be considered flag bearers of a drive towards sustainability that aims to increase the use of earth abundant metals in important stoichiometric and catalytic transformations to ease demand for precious transition metals which have long dominated this chemistry. However, notable in their review is the comparative dearth of isolable, well‐defined hydrides of the alkali metals[^2a^, ^2b^, ^2c^, ^2d^, ^2e^, ^2f^, ^2g^, ^2h^] and total lack of complete group one sets, from the otherwise wide selection of representative metal hydrides. The binary alkali metal hydrides “M^+^H^−^” are salts having cubic rock‐salt lattices with high enthalpy that renders them essentially insoluble in common organic solvents.[^3a^, ^3b^] Valency limitations dictate that bulky polydentate monoanionic ligands such as β‐diketiminates cannot be utilized in the same way for group 1 hydrides as that used in stabilizing group 2 hydrides.[^4a^, ^4b^, ^4c^, ^4d^, ^4e^, ^4f^, ^4g^, ^4h^, ^4i^, ^4j^, ^4k^]

An alternative strategy towards soluble alkali metal hydrides involves the co‐complexation of an alkali metal hydride with another molecule/s. One classic example of such an approach can be found in lithium aluminium hydride (LiAlH_4_) (formally a co‐complex of LiH and AlH_3_), which has long been an indispensable synthetic tool in stoichiometric reduction chemistry.^[5]^ More recently, LiAlH_4_ has found use as a pre‐catalyst in the hydrogenation of imines as shown by Harder and co‐workers.[^6a^, ^6b^] In general, a distinction should be made between such aluminate derivatives and their parent LiH components since the greater covalency and strength of Al−H bonds leads to lower reactivity than their parent lithium hydride components which carry more charged hydride ligands.

**Figure 1:**
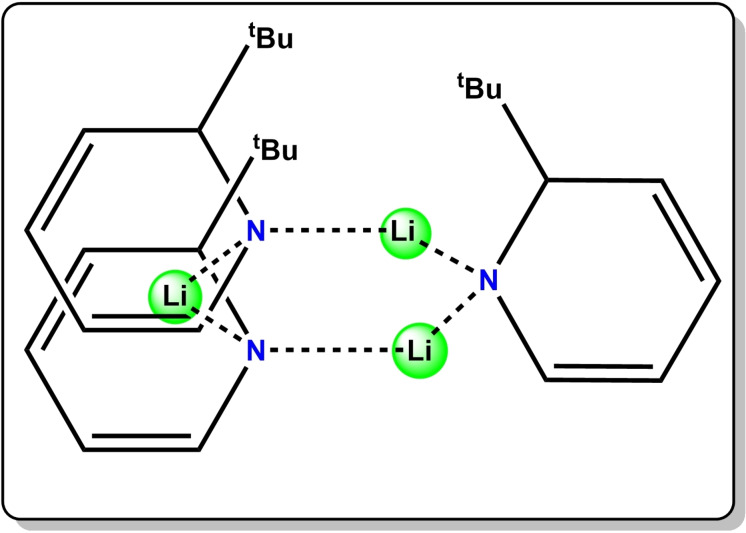
Trimeric aggregation of lithium hydride surrogate 2‐^t^BuC_5_H_5_NLi [Li(^t^BuDHP)] as determined by DOSY NMR studies.

In 2015, our group reported another strategy, wherein a saturated C−H bond in an alkyldihydropyridyl anion bound to lithium in [1‐lithio‐2‐tert‐butyl‐1,2‐dihydropyridine, (2‐^t^BuC_5_H_5_NLi) or for brevity Li(^t^BuDHP)] (Figure 1) can act as a surrogate source of molecular lithium hydride (“LiH”), which is generated concomitantly as the dihydropyridyl unit transforms to an aromatic substituted pyridine. Evidence of the hydridic nature of Li(^t^BuDHP) was first established from the stoichiometric reduction of benzophenone by the “LiH” surrogate.[^7a^, ^7b^] One major property of Li(^t^BuDHP) is its solubility in hexane that has been exploited in homogeneous hydroboration catalysis of aldehydes and ketones and in the dehydrogenative cyclisation of diamine boranes.[^8a^, ^8b^, ^8c^] Encouraged by these initial successes, and aware that alkali metal mediation can be dependent on the specific alkali metal employed,[^9a^, ^9b^, ^9c^, ^9d^, ^9e^] heavier alkali metal *tert*‐butyldihydropyridines [Na(^t^BuDHP) and K(^t^BuDHP)] were synthesised through direct salt metathesis of Li(^t^BuDHP) with sodium and potassium *tert*‐butoxide [Na(O^t^Bu)/K(O^t^Bu)] respectively. Thermal Volatility Analysis (TVA) of the resultant samples showed that the propensity of hydride expulsion of the AM(^t^BuDHP) complexes increased down the group from lithium to potassium, following the trend of decomposition of saline hydrides.^[10]^ However, this increase in reactivity was offset by the loss in solubility of the bulky dihydropyridines in hydrocarbon solvents. We envisaged that the co‐complexation strategy, as stated earlier, might prove to be an apt solution to the solubility problem. By virtue of being the third most abundant element in the Earth's crust,^[11]^ aluminium seemed the perfect choice among main group metals for partnering an alkali metal in a co‐complex.

Di‐iso‐butylaluminium‐2,2,6,6‐tetramethylpiperidide [(^i^Bu)_2_Al(TMP)] has a track record in stabilizing a range of organolithium components to form heterobimetallic ‘ate’ complexes. These ates have had an important role to play in the development of bimetallic cooperativity.[^9c^, ^12^] Prior studies on the reactivity of Li(TMP) and [(^i^Bu)_2_Al(TMP)] in tandem have ascertained that the sterically encumbered TMP ligands allow the lithium amide to display its basic function by deprotonation of an aryl/alkyl substrate, while the organoaluminium moiety traps the new, less hindered organolithium molecule in a method labelled as “trans‐metal‐trapping” (*TMT*).[^13a^, ^13b^, ^13c^, ^13d^, ^13e^, ^13f^, ^13g^] Inspired by this success with Li(TMP), a common amido base, we sought to investigate the possibility of using lithium dihydropyridine [Li(^t^BuDHP)], an amide carrying a surrogate hydride, as an alternative to Li(TMP). From a structural perspective this would open up a new avenue to isolate rarer aluminates of heavier alkali metals. Hence, our target here was to synthesise and characterise a new class of ate compound combining the complete set of alkali metal dihydropyridines with the dialkylamidoaluminium reagent [(^i^Bu)_2_Al(TMP)], in effect heteroleptic alkali metal aluminium hydride surrog[ATES].

## Results and Discussion

**Scheme 1:**
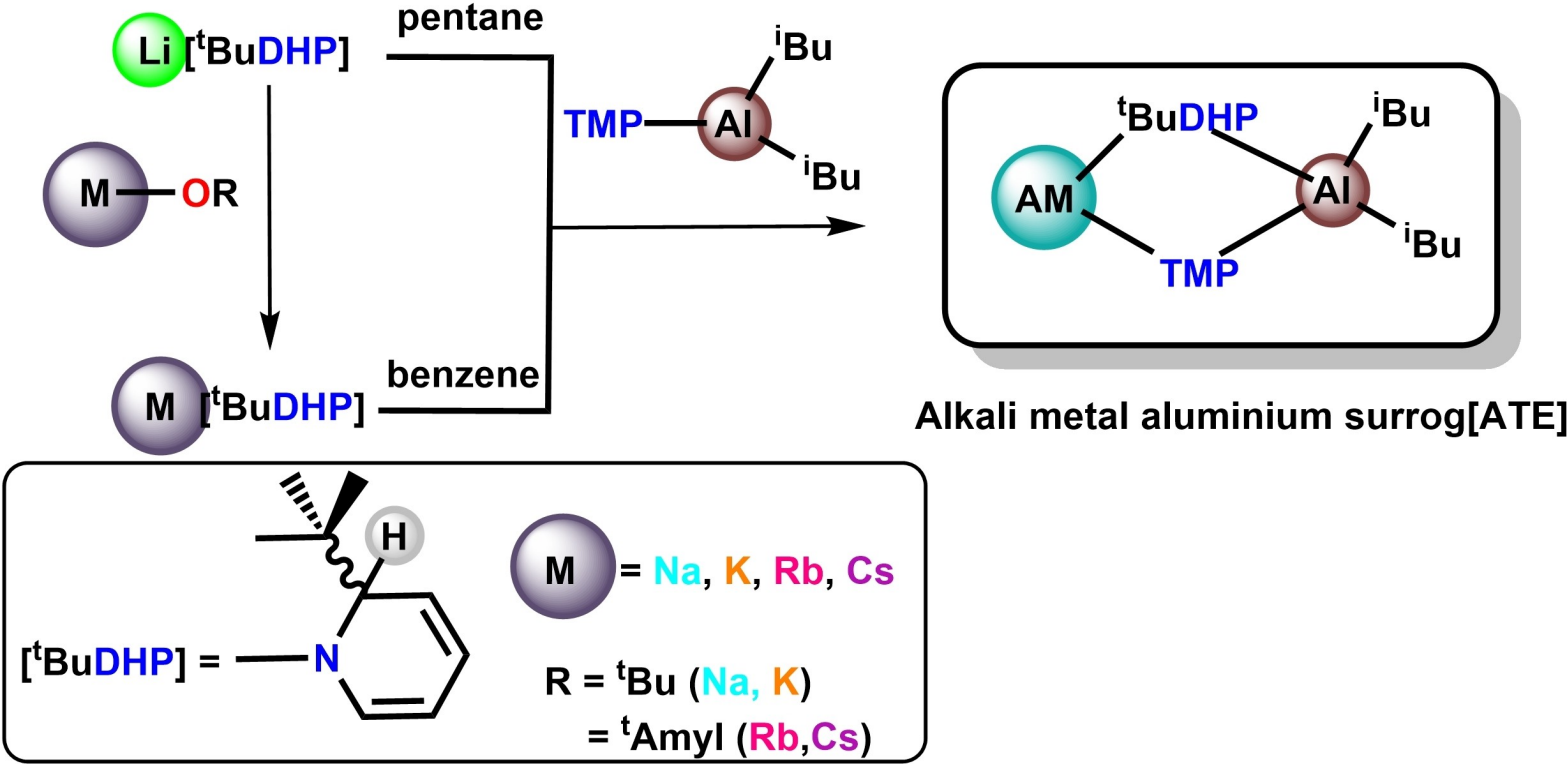
General scheme for preparation of alkali metal aluminium surrog[ATES] by co‐complexation. AM represents the complete alkali metal group (Li‐Cs).

**Figure 2:**
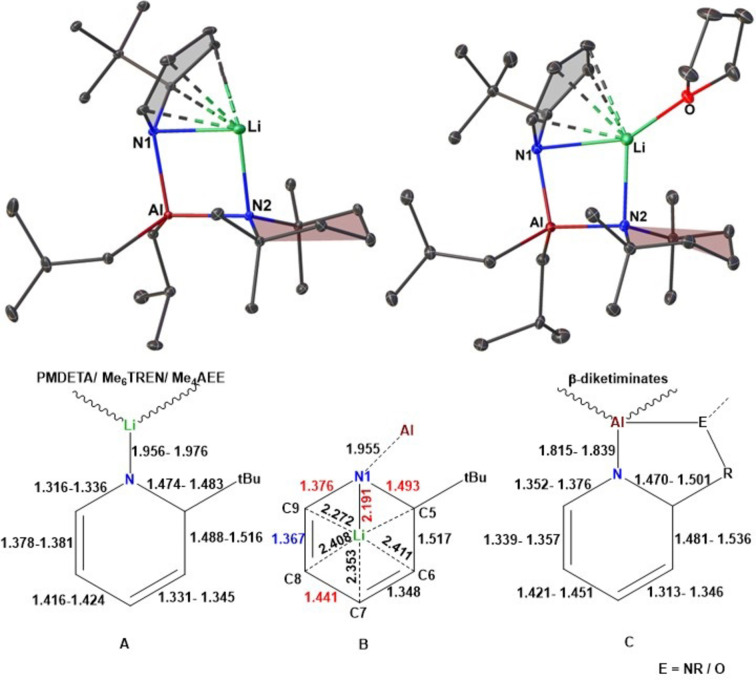
(Top) Molecular structure of [Li(^t^BuDHP)(TMP)Al(^i^Bu)_2_] (**1**) and [(THF)Li(^t^BuDHP)(TMP)Al(^i^Bu)_2_] (**1 a**). All hydrogen atoms have been omitted for clarity. Thermal ellipsoids are displayed at 30 % probability. (Bottom) Comparison of selected bond lengths (Å) in **A**: (Me_4_AEE/ PMDETA/ Me_6_TREN)Li(^t^BuDHP), **B**: Li(^t^BuDHP)(TMP)Al(^i^Bu)_2_ (**1**), and **C**: β‐diketiminate stabilized 1,2‐dihydropyridylaluminium.

Pleasingly, it was found that when an equivalent amount of [(^i^Bu)_2_Al(TMP)] was subjected to a suspension^[14]^ of the lithium hydride surrogate [Li(^t^BuDHP)] in n‐pentane, the resultant mixture turned homogeneous at room temperature (Scheme 1). NMR spectroscopic studies in C_6_D_6_ solution hinted at the formation of a new species as evident from a definite shift in the dihydropyridyl C−H resonances in comparison to those in free Li(^t^BuDHP) (See Supporting Information, Figure S9). Meanwhile, a concentrated solution of the above mixture in n‐pentane cooled to −20 °C overnight yielded colourless crystalline blocks. Single crystal X‐ray analysis of the crystals unveiled a solvent‐free monomer of a bimetallic ate complex, [Li(^t^BuDHP)(TMP)Al(^i^Bu)_2_] (**1**) (Figure 2‐top, left), which crystallized in the P‐1 space group. While the aluminium centre occupies a distorted tetrahedral (NNCC) site, the lithium atom is displaced from its original position on the dihydropyridyl anion. Lithium now prefers to coordinate to the nitrogen atom of the bridging TMP anion and finds itself tucked under the dihydropyridyl ring to take advantage of the π‐surface provided by the conjugated double bonds of the dearomatized ring unlike the donor solvated Li(^t^BuDHP) analogues [(Me_4_AEE)Li(^t^BuDHP), (PMDETA)Li(^t^BuDHP), (Me_6_TREN)Li(^t^BuDHP) where Me_4_AEE=bis‐[2‐(*N*,*N*‐dimethylamino)ethyl]ether, PMDETA=*N*,*N*,*N′*,*N′′*,*N′′*‐pentamethyldiethylenetriamine, and Me_6_TREN=tris[2‐(dimethylamino)ethyl]amine].^[7b]^ A comparable metal⋅⋅⋅metal co‐operative effect was seen to be operative in a previous study by our group, where the participation of Lewis acidic zinc in the pyrrolyl sodium complex [Na(NC_4_H_4_)]_*n*_ allows the alkali metal to modify its bonding nature towards the anionic N‐heterocyclic ligand from σ‐bonding in [(PMDETA)Na(NC_4_H_4_)]_2_ to π‐bonding in the corresponding lower‐order and higher‐order zincate derivatives [{(THF)_2_NaZn(THF)(NC_4_H_4_)_3_}, {(TMEDA)Na}_2_{Zn(NC_4_H_4_)_4_}, and {(PMDETA)Na}_2_{Zn(NC_4_H_4_)_4_} where TMEDA=*N*,*N*,*N′*,*N′*‐tetramethylethylenediamine].^[15]^ The separation between lithium and the unsaturated carbon atoms of the dihydropyridyl moiety in **1** fall in the range of 2.272(3) Å to 2.411(3) Å. The sp^3^ hybridized ring carbon is slightly elevated from the conjugated plane lying at a distance of 2.705(3) Å from lithium. The alternating arrangement of short and long bond lengths within C6−C7−C8−C9−N1 (Figure 2‐bottom‐B) confirms the presence of conjugation in the ^t^BuDHP ring. However, upon comparing the absolute values of the bond parameters we find a close resemblance to β‐diketiminate stabilized 1,2‐dihydropyridylaluminium systems[^16a^, ^16b^] rather than the monometallic lithium analogues^[7b]^ (Figure 2‐bottom). This indicates the preferential bias of the ^t^BuDHP nitrogen atom towards sharing its electron density with the neighbouring aluminium centre. It is also validated by the longer N1−Li distance of 2.191(3) Å in contrast to those in the solvated structures of Li(^t^BuDHP) reported earlier by our group [1.956(8) Å in (Me_4_AEE)Li(^t^BuDHP); 1.976(2) Å in (Me_6_TREN)Li(^t^BuDHP)].^[7b]^ While compound **1** is related to some of the diamido‐dialkyl lithium aluminate examples in the literature^[13d]^ consisting of a similar four‐membered Li−N−Al−N bridging motif, **1** is unusual being a structurally well‐defined example of a donor‐free monomeric ate featuring π‐stabilization.

Addition of THF to the hexane suspension of a mixture containing equimolar amounts of Li(^t^BuDHP) and (^i^Bu)_2_Al(TMP) and subsequently cooling to −30 °C, generated another set of colourless crystals, albeit in poor yield. In this instance, X‐ray crystallographic analysis confirmed the formation of a THF solvated co‐complex [(THF)Li(^t^BuDHP)(TMP)Al(^i^Bu)_2_] (**1 a**) (Figure 2‐top, right).

It may be assumed that Li(^t^BuDHP) cannot be cast as a substitute to Li(TMP) since one decisive criterion for a *TMT* system states that the lithium and aluminium species should not co‐complex with each other to any significant extent to form an ate in order to display unique reactivity profiles. The lower steric profile of Li(^t^BuDHP) rules it out of consideration as a partner in trans‐metal‐trapping chemistry as it readily co‐complexes with the organoaluminium moiety. However, the formation of **1** and **1 a** can be treated as among the first illustrations of a trapped amido anion with a surrogate hydride as opposed to the trapped carbanions in all previous examples.

**Scheme 2:**
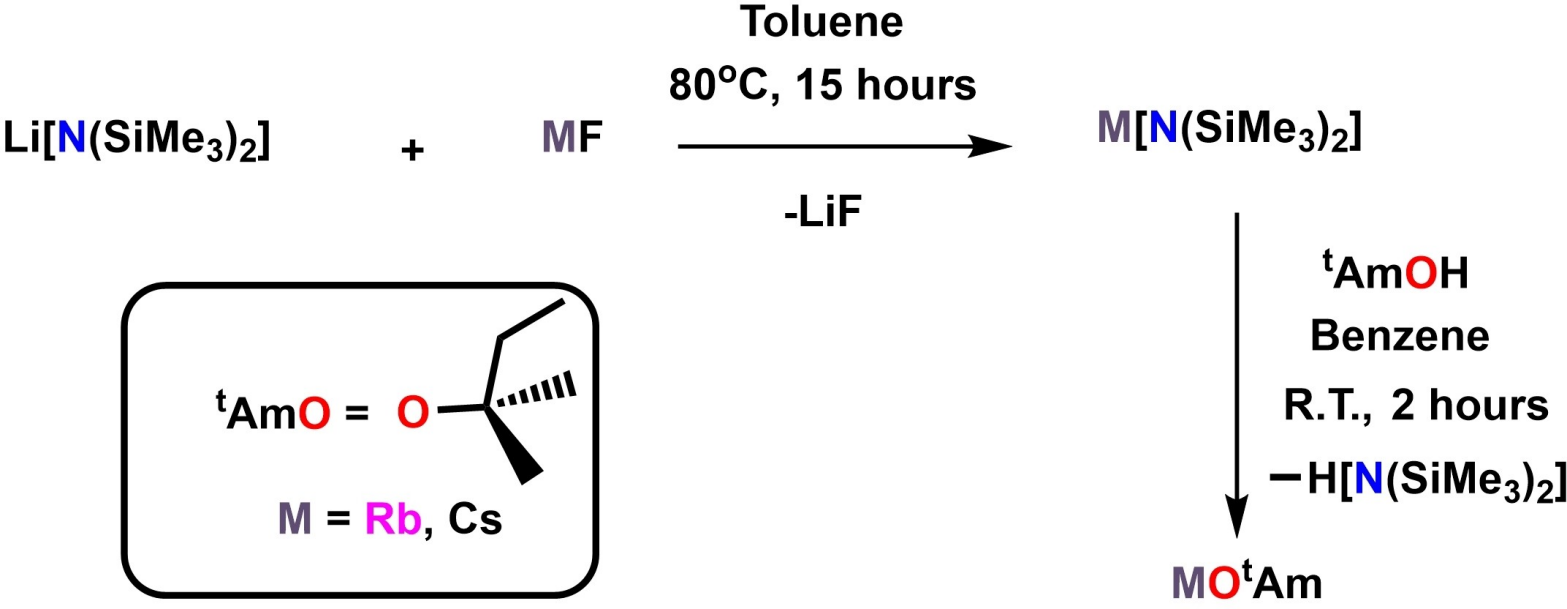
Synthesis of rubidium and caesium alkoxides [Rb‐O^t^Am and Cs‐O^t^Am].

On the back of the progress made with Li(^t^BuDHP) and having recently reported rubidium and caesium aluminyl compounds,^[17]^ we next looked to probe the effect of these more experimentally challenging heavier alkali metals on the aluminate structure. Consequently, a synthetic protocol was established for the sake of isolating the dihydropyridines of rubidium and caesium. Akin to the transmetallation approach utilized for the preparation of sodium and potassium dihydropyridines, the heavier counterparts were synthesised by the σ‐bond metathesis of respective metal alkoxides [M‐OR] with Li(^t^BuDHP). Owing to the practical difficulties involved in the process of isolating a pure batch of heavy alkali metal tert‐butoxide,[^18a^, ^18b^] a benign method avoiding the use of the heavy elemental metal was employed. Reaction of rubidium and caesium 1,1,1,3,3,3‐hexamethyldisilazide [Rb(HMDS) and Cs(HMDS)]^[19]^ with *tert*‐amylalcohol [(Me)_2_C(OH)(Et), ^t^AmOH] in benzene provided the corresponding alkali metal alkoxides [Rb−O^t^Am and Cs−O^t^Am], obtained as white powders upon evacuation of the solvent and other volatiles under reduced pressure (Scheme 2). Treatment of equimolar amounts of these metal alkoxides with Li(^t^BuDHP) in benzene at room temperature allowed the precipitation of unsolvated hydride surrogates of rubidium [Rb(^t^BuDHP)] and caesium [Cs(^t^BuDHP] in quantitative yields. Each compound can be stored under inert conditions as a solid powder after washing away any lithium impurities with benzene. The identities of the above compounds were confirmed by multinuclear NMR spectroscopy (See Supporting Information for NMR characterisation). The dihydropyridyl signals in the ^1^H NMR spectra were in the range of 3.2 ppm to 6.7 ppm and comparable to those of the lighter congeners.^[10]^ The hydrogen atom bonded to the saturated ring carbon can be detected as a doublet at a chemical shift of 3.19 and 3.31 ppm for Rb(^t^BuDHP) and Cs(^t^BuDHP) respectively in deuterated THF. Following the solubility trend of alkali metal dihydropyridines, Rb(^t^BuDHP) and Cs(^t^BuDHP) were found to be insoluble in hydrocarbon solvents. A possible explanation for this observation is that with increase in atomic radii down the group, the tendency to form higher order oligomers/polymers also intensifies.

**Figure 3:**
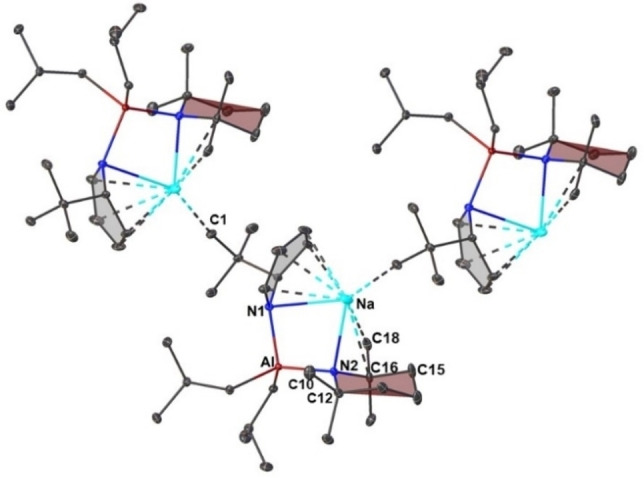
Section of the polymeric structure of [Na(^t^BuDHP)(TMP)Al(^i^Bu)_2_]_∞_ (**2**). All hydrogen atoms have been omitted for clarity. Thermal ellipsoids are displayed at 30 % probability. Symmetry operation to generate equivalent atoms denoted: 1‐x, 1/2+y, 1/2‐z.

Benzene was found to be a suitable medium for [(^i^Bu)_2_Al(TMP)] to combine with the heavier members of the group one ^t^BuDHPs and remain homogeneous in solution (Scheme 1). A concentrated solution of benzene containing Na(^t^BuDHP) and [(^i^Bu)_2_Al(TMP)] produced colourless blocks of crystals after leaving the mixture with slow evaporation of hexane at room temperature. The structure of the compound was determined by single crystal X‐ray diffraction studies and identified as [Na(^t^BuDHP)(TMP)Al(^i^Bu)_2_]_∞_ (**2**; Figure 3). Compound **2** crystallized in the P2_1_/c space group as a donor‐free polymer where sodium is sandwiched between the ^t^BuDHP and TMP rings. The bimetallic units are held together by a short Na⋅⋅⋅C1(^t^Bu) contact of 2.977(2) Å from a neighbouring dihydropyridyl anion to construct a polymeric zig‐zag chain. The asymmetric unit of **2** bears a resemblance to **1** by virtue of having a similar bridging alignment [M−N1−Al−N2] with a distorted tetrahedral geometry at the aluminium centre.

**Figure 4:**
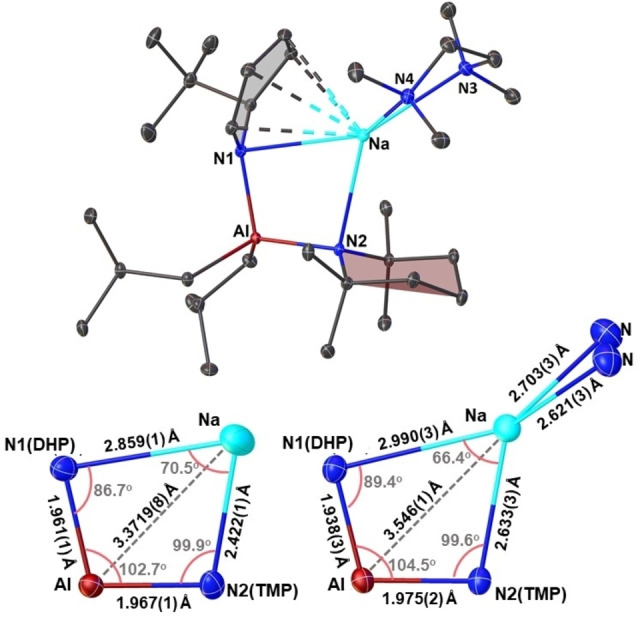
(Top) Molecular structure of [(TMEDA)Na(^t^BuDHP)(TMP)Al(^i^Bu)_2_] (**2 a**). All hydrogen atoms have been omitted for clarity. Thermal ellipsoids are displayed at 30 % probability. (Bottom) Selected bond distances (Å) and bond angles (°) of the four‐membered (Na−N1−Al−N2) ring of **2** (left) and **2 a** (right). Carbon and hydrogen atoms have been omitted for clarity.

When an equivalent amount of TMEDA was added to a benzene solution of **2** formed in situ, crystals of the donor stabilized monomeric sodium aluminate [(TMEDA)Na(^t^BuDHP)(TMP)Al(^i^Bu)_2_] (**2 a**) were formed (Figure 4‐top). Interestingly, the crystal studied is enantiopure with the chiral space group P2_1_, suggesting the material is a conglomerate with a physical mixture of oppositely handed crystals. In the structure, the available lone pairs on the chelating donor diminishes the Lewis acidity of the sodium atom. As a consequence, the separation between Na−N1 and Na−N2 atoms increase (Figure 4‐bottom). This elongation between the sodium and the nitrogen bridges is compensated by shorter Al−N(amido) linkages. Interestingly, the distance between the sodium atom and the TMP nitrogen (Na−N2) in the unsolvated polymer **2** falls within a comparable range to our earlier reported structure of a donor‐supported sodium alkyl‐amido(TMP) aluminate [(TMEDA)Na(TMP)(^i^Bu)Al(^i^Bu)_2_] (Table 1).^[20]^ This ensures an overall close proximity of the saturated TMP ring towards sodium, as a result of which short contacts with the hydrogen atoms (located and refined) on the TMP methyl arms (C18 and C10) and an axial one on the β‐carbon atom (C15) in the range of 2.36(2) Å to 2.64(2) Å can be observed in **2**. Solution studies on the crystals of **2** in C_6_D_6_ confirmed the above observation as the β‐CH_2_(TMP) protons were found to be split into three separate multiplets in the ^1^H NMR spectrum (2H, 1H, and 1H respectively), of which two protons appear shielded at −0.02 (1H) and −0.30 ppm (1H) respectively (See Supporting Information, Figure S14) indicating the close interaction of the sodium centre to the axial H‐ atoms of the TMP moiety in the donor‐free aluminate. The structures of **2** and **2 a** bear significance on the grounds of the paucity of structures of bisamido bridged sodium aluminates with only a few crystallographically characterised examples present in the literature from the Fedyushkin and Chivers groups.[^21a^, ^21b^]

**Table 1:** Selected bond lengths (Å) of **1**, **2**, **2 a**, and [**(TMEDA)Na(TMP)(^i^Bu)Al(^i^Bu)_2_**].^[20]^

**A.M**.	**(TMP)**	**1**	**2**	**2 a**	**[(TMEDA)Na(TMP)(^i^Bu)Al(^i^Bu)_2_]**
Li/Na	N2	2.025(3)	2.422(1)	2.633(3)	2.437(2)
	C10	2.857(3)	3.203(2)	3.281(5)	3.253(3)
	C12	2.809(3)	3.132(2)	3.302(4)	3.185(3)
	C16	2.907(3)	3.008(2)	3.253(3)	3.057(3)
	C18	3.048(3)	3.018(2)	3.210(3)	2.991(3)

**Figure 5:**
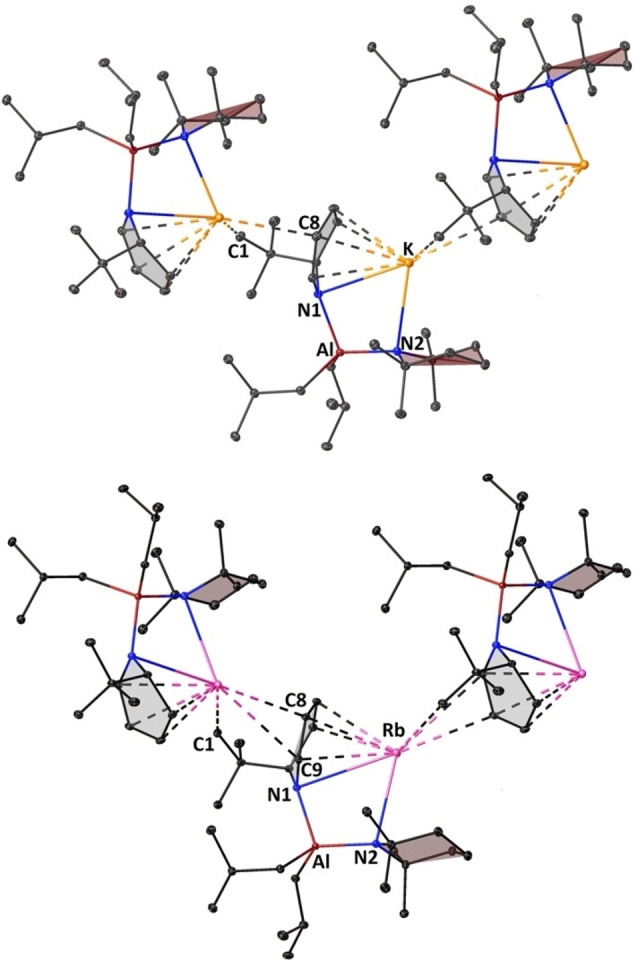
Section of the polymeric structure of (top) [K(^t^BuDHP)(TMP)Al(^i^Bu)_2_]_∞_ (**3**), and (bottom) [Rb(^t^BuDHP)(TMP)Al(^i^Bu)_2_]_∞_ (**4**). All hydrogen atoms have been omitted for clarity. Thermal ellipsoids are displayed at 30 % probability. Symmetry operation to generate equivalent atoms denoted: 1‐x, 1/2+y, 1/2‐z (for **3**) and 1‐x, −1/2+y, 3/2‐z (for **4**).

Our group has previously reported the structural outcomes when different amido groups are present in a series of mono‐amido tris‐alkyl potassium aluminates [(PMDETA)K(μ‐amide)(μ‐^i^Bu)Al(^i^Bu)_2_] [where amide=TMP, DMP (*cis*‐2,6‐dimethylpiperidide) or HMDS complexes].[^22a^, ^22b^] However, attempts to prepare the bis‐amido analogue with K(TMP) and [(^i^Bu)_2_Al(TMP)] induced the deprotonation of one α‐CH_3_ of a TMP unit to give the monomer [(TMEDA)K(μ‐TMP*)(μ‐^i^Bu)Al(^i^Bu)], where TMP* represents a CH_3_‐ and NH‐deprotonated dianionic variant of TMP.^[23]^ Pleasingly, we discovered that reduction of the steric environment to K(^t^BuDHP) produced the first donor free bis‐amido potassium aluminate in [K(^t^BuDHP)(TMP)Al(^i^Bu)_2_]_∞_ (**3**) in a 74 % yield. Similarly, crystals from a Rb−Al combination in benzene were obtained in a yield of 75 % after removing the solvent *in vacuo* followed by adding hexane to the resultant yellow oil at room temperature. Aluminates **3**, and [Rb(^t^BuDHP)(TMP)Al(^i^Bu)_2_]_∞_ (**4**) (Figure 5) crystallized in the same space group as that of **2** and are isostructural and isomorphous in nature. Crystallographically characterised compounds containing both rubidium and aluminium in a bimetallic complex are rare in the literature.[^24a^, ^24b^, ^24c^, ^24d^, ^24e^, ^24f^]

**Figure 6:**
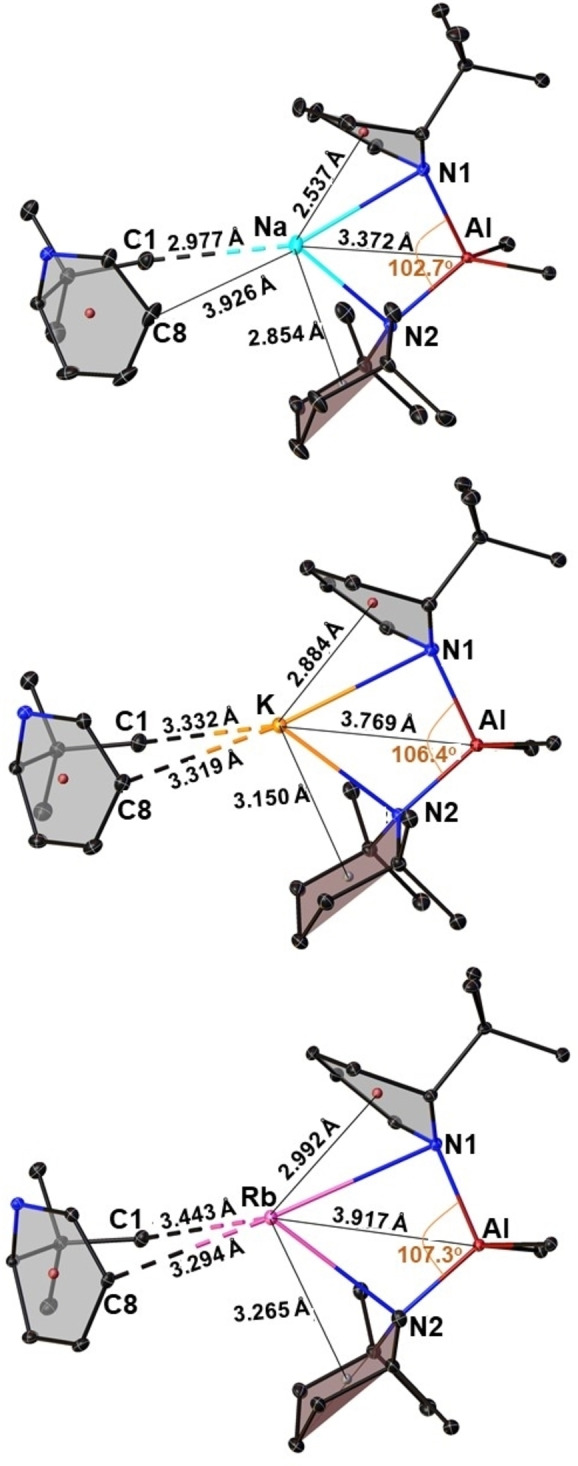
Selected bond distances (Å) and bond angles (°) in the molecular units of the polymeric structures of **2**, **3**, and **4** depicting π‐philicity of heavier alkali metals. Carbon atoms from the ^i^Bu substituent and hydrogen atoms have been omitted for clarity. Thermal ellipsoids are displayed at 30 % probability.

With increasing size of the alkali metal, the Al⋅⋅⋅AM diagonal stretches outwards pushing the centroids of the amido rings further away from the softer metal (Figure 6). This allows room for the neighbouring ^t^BuDHP ring to interact with the larger electropositive alkali‐metal atom. Inspection of the distances between the alkali metal and the carbon atoms of the neighbouring ^t^BuDHP unit revealed that the heavier metals prefer to stay in close contact with unsaturated ring carbon (C8), which lies opposite to the sp^3^ carbon (C5) atom [3.926 Å in **2**>3.319 Å in **3**>3.294 Å in **4**]. Contrary to the above observation, it is found that the distances between neighbouring methyl groups (C1) on the ^t^Bu substituent and the alkali metal follows a reverse order (2.977 Å in **2**<3.332 Å in **3**<3.443 Å in **4**, Figure 6). This points to the increase in the extent of π‐philicity on moving down Group one.[^9d^, ^25^] Another interesting structural modification attributed to the incoming ^t^Bu group can be detected in **2** where Na tends to slip towards C18 and C16 carbons of the TMP ligand as opposed to C12 and C10 in case of lithium in **1**, despite having a similar structural arrangement (See Supporting Information, Figure S36). While Li prefers to sit ‘trans’ to the ^t^Bu group of the ^t^BuDHP, Na likes to rest itself on the site ‘cis’ to the ^t^Bu group of the ^t^BuDHP ligand. This is evident from the contrasting CH_3_(TMP)⋅⋅⋅AM contacts (Table 1). Compounds **3** and **4** show no such preferences as can be ascertained from their comparable distances between the terminal carbon atoms of the TMP chair with respect to the alkali metal.

**Figure 7:**
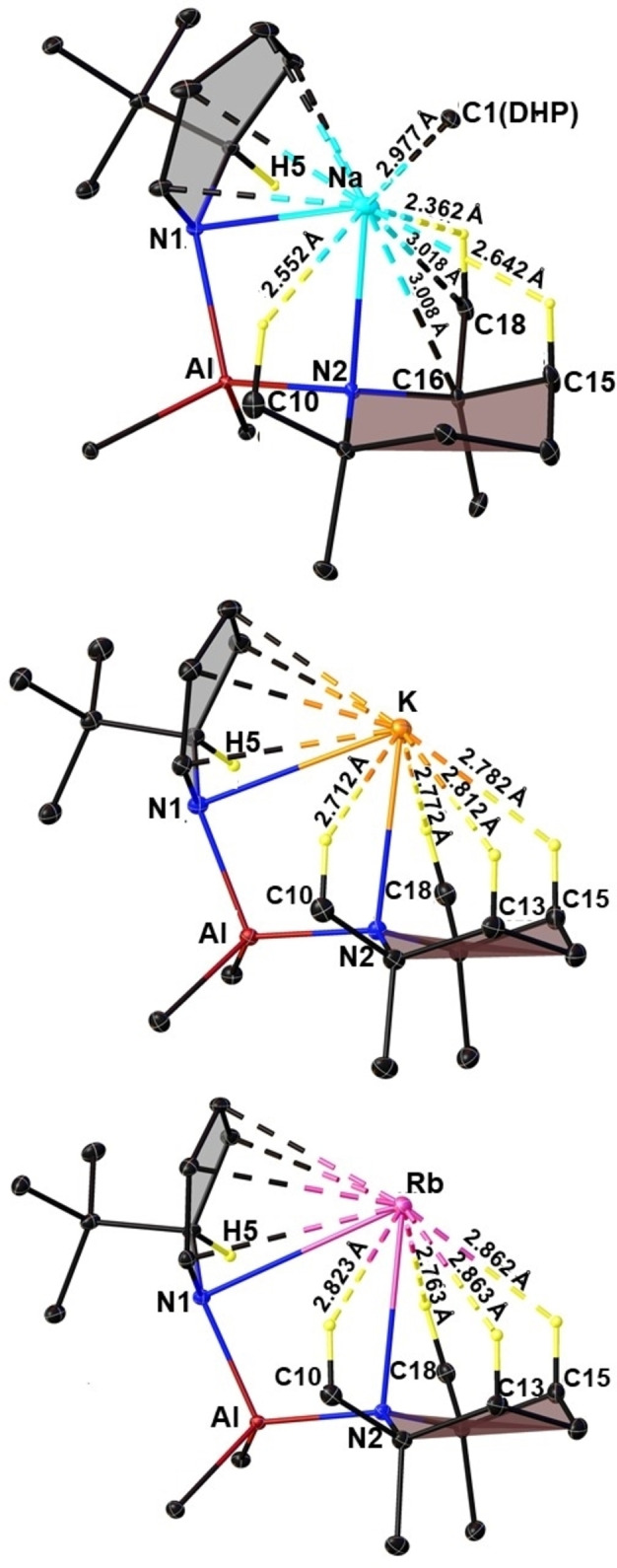
Asymmetric unit of the structures of compounds **2**, **3**, and **4** depicting AM⋅⋅⋅H−C short contacts (Å). Carbon atoms from the ^i^Bu substituent as well as other hydrogen atoms have been omitted for clarity. Thermal ellipsoids are displayed at 30 % probability.

The π‐stabilization offered by the ^t^BuDHP rings to the softer metals coupled with the steric bulk of the ^i^Bu substituents on the aluminium atom brings the axial β‐hydrogen atoms of the saturated TMP ring in close proximity to the alkali metal. This effect is paramount for metals at the bottom of group one. Figure 7 displays the short contacts (less than the sum of the respective van der Waal's radii) between the saturated TMP ring with Na, K, and Rb in compounds **2**, **3**, and **4** respectively. A similar range in the values of C−H⋅⋅⋅Na/K contacts was found in the donor stabilized alkyl amido aluminates of sodium and potassium described previously by our group [2.398 Å–2.924 Å in {(TMEDA)Na(TMP)(^i^Bu)Al(^i^Bu)_2_}^[20]^ and 2.625 Å–2.973 Å in {(PMDETA)K(μ‐amide)(μ‐^i^Bu)Al(^i^Bu)_2_}].^[22a]^ The C−H (TMP)⋅⋅⋅Rb contacts in **4** are distinctively shorter than the ones reported earlier by our group in the homoleptic TMEDA solvate [(TMEDA)Rb(TMP)]_2_.^[26]^ Although there have been a few studies on anagostic C−H⋅⋅⋅Li interactions in diorgano lithium aluminates,[^27a^, ^27b^, ^27c^] the field of alkyl amido alkali metal aluminates is still unexplored.

While the formation of a co‐complexation product from a mixture of Cs(^t^BuDHP) and [(^i^Bu)_2_Al(TMP)] in benzene solution could be proved from the comparable ^1^H NMR resonances (See Supporting Information, Figure S31) against those of its lighter siblings, attempts to crystallize the same from its oil were unsuccessful. Addition of hexane to the oily substance resulted in the formation of a sticky solid. The presence of a variety of nitrogen and oxygen donors such as PMDETA, TMEDA, THF or 18‐crown‐6‐ether in a hexane suspension of the bimetallic mixture failed to produce crystalline samples suitable for X‐ray crystallographic study. However, the solubility of the compound in benzene, points to the effectiveness of the aluminium agent in minimizing the oligomerization of the surrogate caesium hydride.

It has been well documented that attractive London Dispersion Forces (LDF) influence the stability, structure, and reactivity of organometallic compounds.[^28a^, ^28b^] Lately, these non‐covalent interactions have been found to be a factor towards the stabilization of theoretically calculated intermediates for bimetallic potassium‐aluminyl compounds,[^29a^, ^29b^] exhibiting interesting alkali‐metal‐mediated reactivity.[^30a^, ^30b^] A recent study by Power and co‐workers demonstrated the significance of LDF in the incomplete reduction of 1‐adamantol with alkali metals (Li, Na, and K).^[31]^ Having a series of group one aluminate structures at our disposal, we sought to quantify the interactions in the unsolvated complexes **1**, **2**, **3**, and **4** into electrostatic and dispersive components. Attractive dispersion interactions are an important intermediate range class of stabilizing interactions in organometallic complexes. In order to compare the magnitude of anagostic bond character in four metal ion complexes we optimized a monomeric unit within each crystal structure using the B3LYP[^32a^, ^32b^] functional and def2‐TZVP^[33]^ basis set with the auxiliary basis def2/J.^[34]^ We used the built‐in D4[^35a^, ^35b^] algorithm to evaluate dispersion as it is more accurate for ionic metal systems than the earlier models (D3/D3BJ)^[36]^ due to on‐the‐fly computation of atomic polarizabilities which can account for changes in the underlying electron densities and adjust dipole‐dipole dispersion coefficients (C_6_). The electrostatic interaction energy was calculated according to Equation (1). We minimized basis set superposition error (BSSE) by calculating a counterpoise corrected interaction energy which increased electronic interaction energies of these four complexes by an average of 32 kJ mol^−1^.^[37]^

(1) $$E_{interaction}=E_{complex}-\left(E_{{aluminate}^{-1}}+E_{{metal}^{+1}}\right)$$

$$BSSE=E_{{aluminate-optimized}^{-1}}+E_{aluminateghostatoms^{-1}}+E_{metalghostatoms^{+1}}{-2(E}_{{aluminate}^{-1}})-E_{{metal}^{+1}}$$

$$E_{Counterpoise-corrected-interaction-energy}=E_{interaction}-BSSE$$

**Figure 8:**
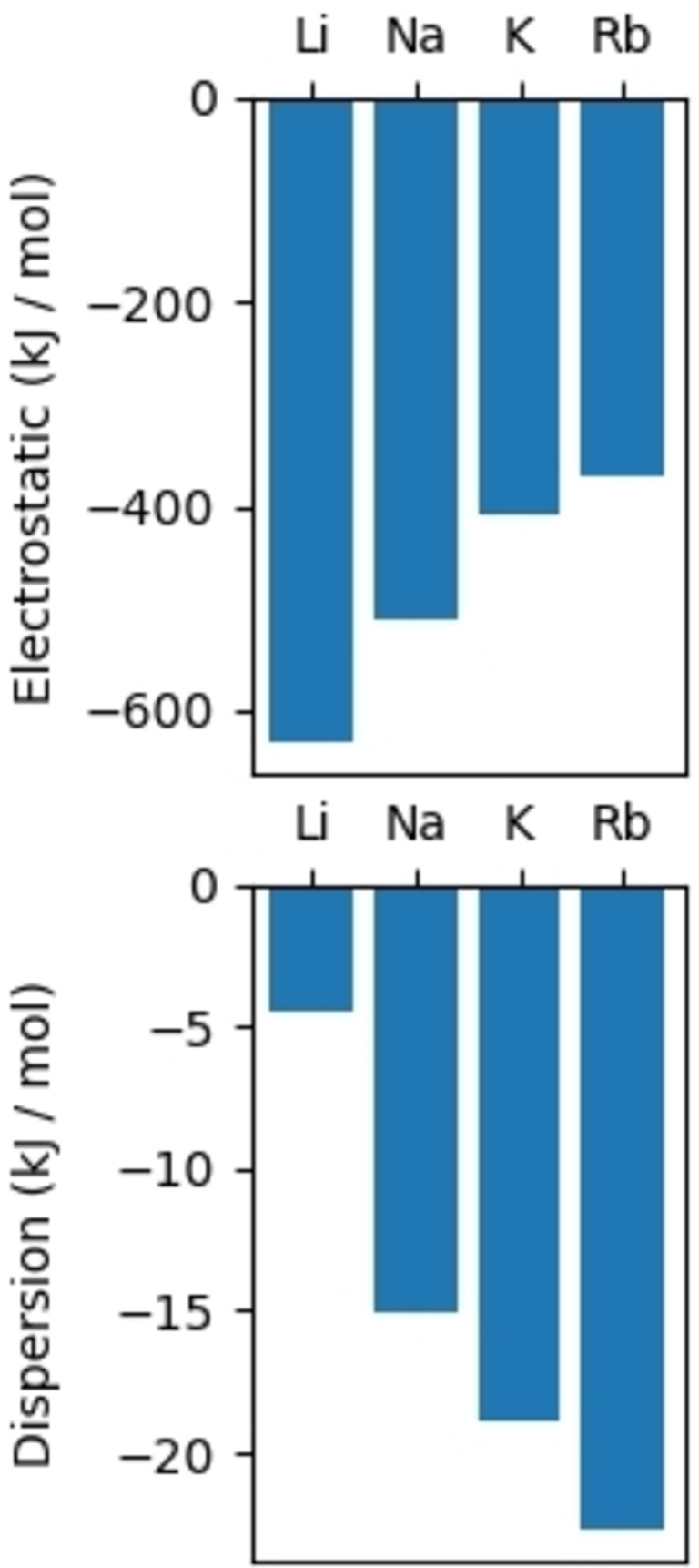
Electrostatic interaction energy and dispersion interaction energy between the alkali metal (Li, Na, K, Rb) and aluminate [(^t^BuDHP)Al(TMP)(^i^Bu)_2_] for compounds **1**, **2**, **3**, and **4** showing a decreasing trend as ion size increases for the former and an increasing trend in dispersion energy for the latter.

The calculations revealed that the alkali‐metal aluminates possess strong interaction energies ranging from approximately −635 kJ mol^−1^ for lithium to −393 kJ mol^−1^ for rubidium. As evident from Figure 8, the electrostatic forces are a major contributor towards the stabilization of these complexes in comparison to dispersive interactions. However, as expected, a trend of decreasing ionic interactions can be observed upon moving down group one [Energy (*E*) (kJ mol^−1^) in order of increasing stability=−630.23 (Li)>−511.65 (Na)>−410.02 (K)>−370.45 (Rb)], which is antithetical to the calculated LDF values [Energy (*E*) (kJ mol^−1^) in order of increasing stability=−4.47 (Li)<−15.07 (Na)<−18.92 (K)<−22.67 (Rb)] (Figure 8). Notably the increase in the LDF is particularly significant at the lithium sodium junction. These results follow a similar pattern to the one reported by Izgorodina and co‐workers in a study focusing on the stability of different isomers of a series of Li−K N‐(α‐methylbenzyl)allylamides.^[38]^ We find that as ion sizes decrease the magnitude of electronic interaction energy decreases due to higher charge density, while dispersion attraction energy increases with ion size due to the distances of the outer electrons to the nuclei.

**Figure 9:**
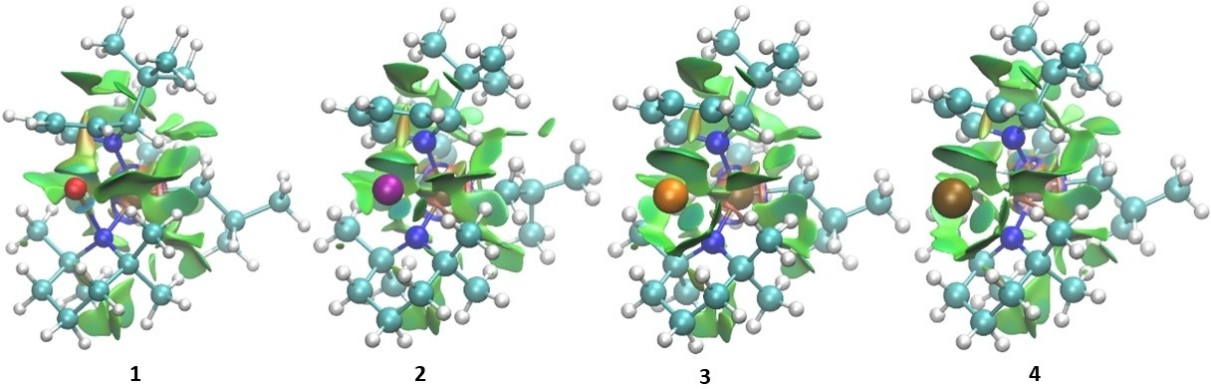
Visualizations of each of the donor‐free complexes (**1**, **2**, **3**, and **4**) described herein with non‐covalent interactions explicitly shown using the NCIPlot software.

In order to better visualize and understand the myriad of non‐covalent interactions (NCI) we used the NCIPlot software with ultrafine integration to generate the representations in Figure 9.[^39^, ^40^] By examining electron density and derivatives thereof it is possible to identify gradient critical points which in real space are visualized as the green isosurface shown. We find as the size of the ion increases so too does the size of the isosurface representing the various NCIs. In order to be able to generate these diagrams the Def2/J effective core potential was disabled to allow for all electrons in the system to be explicitly represented. Both the methyl arms as well as the methylene ring in the TMP ligand provide stability to the alkali metals by virtue of non‐covalent interactions. The stabilizing effect furnished by the methylene ring is pronounced in the case of K and Rb aluminates (**3** and **4**). These results support the existence of the observed short contacts [AM⋅⋅⋅H−C(TMP)] in the donor‐free heavy alkali‐metal aluminates (**3** and **4**). Surprisingly, considering the high utility of TMP and the numerous structures it is found in,^[41]^ the effects of non‐covalent interactions arising from it within structures has been rarely mentioned previously.

We next attempted to study the stability of these complexes in light of the presence of these stabilizing interactions, by heating samples of the donor free ates **1**–**4** at 85 °C in deuterated benzene. This showed that lithium aluminate **1** is the most stable, with no evidence of MH elimination/rearomatized pyridine formation even after heating for 24 h. However, this study provided evidence of metal hydride elimination, as seen by the appearance of the characteristic 2‐*t*‐butylpyridine resonances in the ^1^H NMR spectra (see Supporting Information, Figures S37–S40) over 2–3 days. In order to preliminarily test the surrogate hydride reactivity of these aluminates, experiments with the lightest and heaviest aluminates of the series (Li and Cs) were carried out at room temperature in hexane and benzene solution respectively with stoichiometric amounts of benzophenone. Quenching these mixtures with H_2_O resulted in the precipitation of insoluble hydroxide salts along with the formation of benzhydrol, which was characterised by NMR spectroscopy (see Supporting Information, Figures S41–S46). However, these results do not explain the origin of the hydride source, since a previous report by our group indicated the availability of masked (β) hydride on the isobutyl groups bound to aluminium in the reduction of benzophenone, mimicking a Meerwein‐Pondorf‐Verley type mechanism.^[42]^ To investigate the above possibility we monitored these reactions in a J. Young's NMR tube by adding one equivalent of the ketone to lithium aluminate **1** in C_6_D_6_ at room temperature (see Supporting Information, Figure S47). The appearance of characteristic isobutene resonances (*δ*=4.73 and 1.59 ppm) in the ^1^H NMR spectrum suggests the role of the masked hydride from the isobutyl groups. Addition of a further equivalent of benzophenone to this mixture revealed a complete loss of isobutyl resonances, confirming the aforementioned possibility. To our surprise, addition of a third equivalent of benzophenone at room temperature did not yield any 2‐*t*‐butylpyridine. The dihydropyridyl resonances appear shifted compared to both the starting material and also homometallic [Li(^t^BuDHP)], while the ^7^Li resonance shifts from −2.10 in **1** to 1.90 ppm (see Supporting Information, Figure S48), suggesting an unidentified bimetallic species is formed. This lack of reactivity of the dihydropyridyl moiety suggests that the presence of the aluminium component provides a stabilizing influence since [Li(^t^BuDHP)] itself readily reduces benzophenone with concomitant generation of 2‐*t*‐butylpyridine. When this experiment was replicated with isolated crystals of the rubidium aluminate (**4**), the appearance of both isobutene as well as the 2‐t‐butylpyridine resonances in the ^1^H NMR spectrum were seen, hinting towards increased surrogate hydride reactivity of the heavier group one aluminates (see Supporting Information, Figure S49). Resonances at 6.09 and 6.27 ppm in the ^1^H NMR spectrum allude to the fact that there are potentially two distinct −OC(*H*)Ph_2_ environments in the product which has been witnessed previously in homometallic [(TMP)(Ph_2_(H)CO)Al(μ‐OC(H)Ph_2_)]_2_ which shows distinct bridging and terminal resonances.^[42]^ Taken together this demonstrates a complicated pattern of surrogate/masked hydride reactivity for this family of bimetallic alkali‐metal aluminates.

## Conclusion

In summary, this study has exported alkali metal dihydropyridines [AM(^t^BuDHP)] into the chemistry of aluminates. The dialkyl amido aluminium compound [(^i^Bu)_2_Al(TMP)] plays a fundamental role as a stabilizer by delivering the alkali metal surrogate hydride [AM(^t^BuDHP)] into a hydrocarbon solvent when added in stoichiometric quantities. The crystal structures of [Li(^t^BuDHP)(TMP)Al(^i^Bu)_2_] (**1**), [Na(^t^BuDHP)(TMP)Al(^i^Bu)_2_]_∞_ (**2**), [K(^t^BuDHP)(TMP)Al(^i^Bu)_2_]_∞_ (**3**), and [Rb(^t^BuDHP)(TMP)Al(^i^Bu)_2_]_∞_ (**4**) demonstrated that the dihydropyridyl anion can act as a N‐heterocyclic group with a surrogate hydride providing stability to the alkali metals through π‐donation. These structures are one of the first in a series of donor free ates with two distinct amido bridges. The difference in the aggregation states is reflected in their solubility. While the lithium congener is soluble in an aliphatic hydrocarbon solvent, it takes an aromatic (benzene) solvent to dissolve the other group one aluminates. The polymers can be broken down into monomeric units by the employment of neutral Lewis base donors as observed during the isolation of [(THF)Li(^t^BuDHP)(TMP)Al(^i^Bu)_2_] (**1 a**) and [(TMEDA)Na(^t^BuDHP)(TMP)Al(^i^Bu)_2_] (**2 a**). Energy calculations on the stable donor free complexes provided us with an insight into the nature of alkali‐metal‐ligand interactions where a definite trend in increased contribution of attractive London Dispersion Forces can be observed on moving down group one, which is in sharp contrast to the electrostatic forces of attraction. The NCI plots demonstrated that the methylene groups of a saturated TMP ligand can lend stability to the large cations of group one. Preliminary test reactions suggest that the stability imparted on the dihydropyridyl unit as a consequence of the presence of the organoaluminium fragment modifies its reactivity as a metal hydride source, with the degree of modification depending upon the identity of the alkali metal. Our ongoing work is to investigate further this facet of their behaviour as well as to probe other reactivity scenarios in both stoichiometric and catalytic applications.

## Experimental Section

General experimental procedures and detailed synthetic methods with characterisation of compounds can be found in the Supporting Information. Single crystal diffraction data for **1** (CCDC 2165117), **1 a** (CCDC 2165118), **2** (CCDC 2165119), **2 a** (CCDC 2165120), **3** (CCDC 2165121), **4** (CCDC 2165122) are reported in crystallographic information files (CIF) accompanying this document.

Deposition Number(s) 2165117 (**1**), 2165118 (**1 a**), 2165119 (**2**), 2165120 (**2 a**), 2165121 (**3**), 2165122 (**4**) contain(s) the supplementary crystallographic data for this paper. These data are provided free of charge by the joint Cambridge Crystallographic Data Centre and Fachinformationszentrum Karlsruhe Access Structures service.

Full details on data collection, reduction and refinement can be found in the individual CIFs.

### Synthesis of selected compounds

**Synthesis of Li(^t^BuDHP)(TMP)Al(^i^Bu)_2_ (1)**: Li(^t^BuDHP)^[7a]^ (0.143 g, 1 mmol) was suspended in 3 mL of dry n‐pentane inside a clean dry Schlenk flask and ^i^Bu_2_AlTMP^[43]^ (0.281 g, 1 mmol) was added to it. The mixture was stirred for 1 h at room temperature resulting in a pale‐yellow solution. Upon concentrating and storing the solution at −20 °C colourless blocks of crystals were obtained overnight. Yield=0.298 g, 70 %. Elemental analysis: Calculated values for C_26_H_50_AlLiN_2_ (424.61 g mol^−1^): C 73.54, H 11.87, N 6.60; Found: C 73.50, H 11.82, N 6.43. ^1^H NMR [400.03 MHz, 300 K, C_6_D_12_]: *δ* 0.98 ppm (s, 9H, −^t^Bu[DHP]), *δ* 3.83 ppm (d, 1H, H2[DHP]), *δ* 4.94 ppm (dd, 1H, H3[DHP]), *δ* 5.34 ppm (dd, 1H, H5[DHP]), *δ* 6.27 ppm (dd, 1H, H4[DHP]), *δ* 7.14 ppm (d, 1H, H6[DHP]), *δ* 1.72 ppm (m, 2H, β‐CH_2_[TMP]), *δ* 0.92 ppm (m, 1H, β‐CH_2_[TMP]), *δ* 0.80 ppm (m, 1H, β‐CH_2_[TMP]), *δ* 1.96 ppm (m, 1H, γCH_2_[TMP]), *δ* 1.47–1.54 ppm (m, 12H, −CH_3_[TMP]+1H, γCH_2_[TMP]), *δ* 1.11–1.16 ppm (m, 12H, −CH_3_[^i^Bu]), *δ* 2.13 ppm (m, 2H, −CH[^i^Bu]), *δ* 0.19–0.35 ppm (m, 2H, −CH_2_[^i^Bu]), *δ* 0.49–0.57 ppm (m, 2H, −CH_2_[^i^Bu]); ^13^C {1 H} NMR [C_6_D_12_, 100.60 MHz, 300 K]: *δ* 147.62 ppm (−CH(6)[DHP]), *δ* 123.42 ppm (−CH(4)[DHP]), *δ* 107.16 ppm (−CH(3)[DHP]), *δ* 99.09 ppm (−CH(5)[DHP]), *δ* 59.70 ppm (−CH(2)[DHP]), *δ* 24.76 ppm (−^t^Bu[DHP]), *δ* 40.68 ppm (quaternary[DHP]), *δ* 45.45+46.24 ppm (β‐CH_2_[TMP]), *δ* 17.81 ppm (γCH_2_[TMP]), *δ* 36.69+38.01 ppm (CH_3_[TMP]), *δ* 51.99+51.76 ppm (2× quaternary[TMP]), *δ* 26.73 ppm (−CH[^i^Bu]), *δ* 27.97+28.10 ppm (−CH_3_[^i^Bu]), The resonances of −CH_2_[^i^Bu] could not be observed;^[44]^ ^7^Li (C_6_D_12_, 155.50 MHz, 300 K) *δ*=−1.71 ppm (s).

**Synthesis of [(THF)Li(^t^BuDHP)(TMP)Al(^i^Bu)_2_)] (1 a)**: Li(^t^BuDHP)^[7a]^ (0.143 g, 1 mmol) and ^i^Bu_2_AlTMP^[43]^ (0.281 g, 1 mmol) were added to a clean dry Schlenk flask along with 10 mL of n‐hexane at room temperature. This was left to stir for 10 minutes. To obtain a completely soluble solution, THF was added dropwise. This resulted in a colourless oil forming, from which crystals grew from at −30 °C. Yield=0.078 g, 16 %. Due to the low yield, clean NMR spectra and Elemental Analysis could not be obtained.

**Synthesis of [Na(^t^BuDHP)(TMP)Al(^i^Bu)]_∞_ (2)**: Na(^t^BuDHP)^[10]^ (0.159 g, 1 mmol) was suspended in 3 mL of dry benzene inside a clean dry Schlenk flask and ^i^Bu_2_AlTMP^[43]^ (0.281 g, 1 mmol) was added to it. The mixture was stirred for 1 h at room temperature resulting in a yellow solution. Upon layering the concentrated benzene solution with n‐hexane at room temperature, colourless blocks of crystals were obtained in two days. Yield=0.295 g, 67 %. Elemental analysis: Calculated values for [C_26_H_50_AlNaN_2_] (440.66 g mol^−1^): C 70.87, H 11.44, N 6.36; Found: C 70.80, H 11.36, N 5.98. ^1^H NMR [400.03 MHz, 300 K, C_6_D_6_]: *δ* 1.18 ppm (s, 9H, −^t^Bu[DHP]), *δ* 3.76 ppm (d, 1H, H2[DHP]), *δ* 4.59 ppm (dd, 1H, H3[DHP]), *δ* 4.74 ppm (t, 1H, H5[DHP]), *δ* 5.85 ppm (dd, 1H, H4[DHP]), *δ* 7.11 ppm (d, 1H, H6[DHP]), *δ* 1.35 ppm (m, 2H, β‐CH_2_[TMP]), *δ* −0.30 ppm (t, 1H, β‐CH_2_[TMP]), *δ* −0.02 ppm (t, 1H, β‐CH_2_[TMP]), *δ* 1.73 ppm (m, 1H, γCH_2_[TMP]), *δ* 1.09+1.15+1.39+1.51 ppm (br s, 4× 3H, −CH_3_[TMP]), *δ* 1.09 ppm (br s, 1H, γCH_2_[TMP]), *δ* 1.43–1.47 ppm (m, 12H, −CH_3_[^i^Bu]), *δ* 2.46 ppm (m, 2H, −CH[^i^Bu]), *δ* 0.38–0.87 ppm (m, 4H, −CH_2_[^i^Bu]); ^13^C {1 H} NMR [C_6_D_6_, 100.60 MHz, 300 K]: *δ* 147.65 ppm (−CH(6)[DHP]), *δ* 125.41 ppm (−CH(4)[DHP]), *δ* 105.02 ppm (−CH(3)[DHP]), *δ* 94.96 ppm (−CH(5)[DHP]), *δ* 59.96 ppm (−CH(2)[DHP]), *δ* 25.66 ppm (−^t^Bu[DHP]), *δ* 41.55 ppm (quaternary[DHP]), *δ* 45.27 ppm (β‐CH_2_[TMP]), *δ* 17.72 ppm (γCH_2_[TMP]), *δ* 37.56+38.80 ppm (CH_3_[TMP]), *δ* 27.34+27.51 ppm (−CH[^i^Bu]), *δ* 28.24+28.59+29.27+29.94 ppm (−CH_3_[^i^Bu]), The resonances of −CH_2_[^i^Bu] and quatenary[TMP] could not be observed.

**Synthesis of [(TMEDA)Na(^t^BuDHP)(TMP)Al(^i^Bu)] (2 a)**: Na(^t^BuDHP)^[10]^ (0.018 g, 0.11 mmol) was suspended in 0.5 mL of dry benzene inside a clean dry vial in the glove box and ^i^Bu_2_AlTMP^[43]^ (0.033 g, 0.11 mmol) was added to it. The mixture was stirred for 30 minutes at room temperature resulting in a yellow solution. An equivalent amount of TMEDA (16 μL, 0.11 mmol) was added dropwise and the resultant mixture was layered with n‐pentane. Colourless blocks of crystals were obtained after thirty days at −20 °C. Yield=0.038 g, 63 %. A satisfactory elemental analysis for the bulk material of [(TMEDA)Na(^t^BuDHP)(TMP)Al(^i^Bu)_2_] (2a) was not obtained, which may be attributed to decomposition during shipping and/or sample preparation. Best values are given, nevertheless. Elemental analysis: Calculated values for C_32_H_66_AlN_4_Na (556.86 g mol^−1^): C 69.02, H 11.95, N 10.06; Found: C 68.98, H 11.73, N 7.59. ^1^H NMR [400.03 MHz, 300 K, C_6_D_12_]: *δ* 0.94 ppm (s, 9H, −^t^Bu[DHP]), *δ* 3.74 ppm (d, 1H, H2[DHP]), *δ* 4.70 ppm (dd, 1H, H3[DHP]), *δ* 4.76 ppm (t, 1H, H5[DHP]), *δ* 6.02 ppm (dd, 1H, H4[DHP]), *δ* 7.04 ppm (d, 1H, H6[DHP]), *δ* 2.37 ppm (s, 4H, −CH_2_[TMEDA]), *δ* 2.27 ppm (s, 12H, −CH_3_[TMEDA]), *δ* 1.70 ppm (br s, 2H, γCH_2_[TMP]), *δ* 1.31–1.41 ppm (m, 12H, −CH_3_[TMP]+4H, β‐CH_2_[TMP]), *δ* 1.04 ppm (m, 12H, −CH_3_[^i^Bu]), *δ* 2.06 ppm (m, 2H, −CH[^i^Bu]), *δ* 0.04–0.47 ppm (m, 4H, −CH_2_[^i^Bu]); ^13^C {1 H} NMR [C_6_D_12_, 100.60 MHz, 300 K]: *δ* 147.06 ppm (−CH(6)[DHP]), *δ* 123.98 ppm (−CH(4)[DHP]), *δ* 105.62 ppm (−CH(3)[DHP]), *δ* 93.75 ppm (−CH(5)[DHP]), *δ* 60.21 ppm (−CH(2)[DHP]), *δ* 25.19 ppm (−(CH_3_)_3_[DHP]), *δ* 41.32 ppm (quaternary[DHP]), *δ* 57.91 ppm (−CH_2_[TMEDA]), *δ* 46.73 ppm (−CH_3_[TMEDA]), *δ* 45.83 ppm (β‐CH_2_[TMP]), *δ* 18.16 ppm (γCH_2_[TMP]), δ 26.09 ppm (CH_3_[TMP]), *δ* 51.65 ppm (quaternary[TMP]), *δ* 26.92+27.03 ppm (−CH[^i^Bu]), *δ* 27.97+28.60+29.06 ppm (−CH_3_[^i^Bu]), The resonances of −CH_2_[^i^Bu] could not be observed.

**Synthesis of [K(^t^BuDHP)(TMP)Al(^i^Bu)]_∞_ (3)**: K(^t^BuDHP)^[10]^ (0.129 g, 0.75 mmol) was suspended in 3 mL of dry benzene inside a clean dry Schlenk flask and ^i^Bu_2_AlTMP^[43]^ (0.215 g, 0.75 mmol) was added to it. The mixture was stirred for 1 h at room temperature. resulting in an orange solution. The solvent was evacuated *in vacuo* to form a yellow oil. Upon treating the oil with 2 mL of hexane crystals crashed out immediately from the oil. Yield=0.253 g, 74 %. A satisfactory elemental analysis for the bulk material of [K(^t^BuDHP)(TMP)Al(^i^Bu)_2_] (3) was not obtained, which may be attributed to decomposition during shipping and/or sample preparation. Best values are given, nevertheless. Elemental analysis: Calculated values for [C_26_H_50_AlKN_2_] (456.77 g mol^−1^): C 68.37, H 11.03, N 6.13; Found: C 67.84, H 9.98, N 4.67. ^1^H NMR [400.03 MHz, 300 K, C_6_D_6_]: *δ* 1.25 ppm (br s, 9H, −(CH_3_)_3_[DHP]+6H, −CH_3_[TMP]+2H, β‐CH_2_[TMP]), *δ* 3.79 ppm (d, 1H, H2[DHP]), *δ* 4.54 ppm (t, 1H, H3[DHP]), *δ* 4.67 ppm (dd, 1H, H5[DHP]), *δ* 5.74 ppm (dd, 1H, H4[DHP]), *δ* 7.04 ppm (d, 1H, H6[DHP]), *δ* 1.38 ppm (m, 6H, −CH_3_[TMP]), *δ* 0.87 ppm (m, 1H, β‐CH_2_[TMP]), *δ* 1.47 ppm (m, 12H, −CH_3_[^i^Bu]+2H, γCH_2_[TMP]+1H, β‐CH_2_[TMP]), *δ* 2.50 ppm (m, 2H, −CH[^i^Bu]), *δ* 0.41–0.83 ppm (m, 4H, −CH_2_[^i^Bu]); ^13^C {1 H} NMR [C_6_D_6_, 100.60 MHz, 300 K]: *δ* 147.92 ppm (−CH(6)[DHP]), *δ* 125.67 ppm (−CH(4)[DHP]), *δ* 104.02 ppm (−CH(3)[DHP]), *δ* 95.18 ppm (−CH(5)[DHP]), *δ* 66.28 ppm (−CH(2)[DHP]), *δ* 26.08 ppm (−^t^Bu[DHP]), *δ* 41.78 ppm (quaternary[DHP]), *δ* 18.13 ppm (γCH_2_[TMP]), *δ* 51.50 ppm (quaternary[TMP]), *δ* 27.44+27.73 ppm (−CH[^i^Bu]), *δ* 28.44+28.76+29.63+29.88 ppm (−CH_3_[^i^Bu]). The resonances of −CH_2_[^i^Bu] could not be observed. The resonances of β‐CH_2_[TMP] and −CH_3_[TMP] were not assigned due to overlap of the methyl signals (from TMP and ^i^Bu groups) in ^1^H^13^C‐HSQC NMR spectrum.

**Synthesis of [Rb(DHP)(TMP)Al(^i^Bu)]_∞_ (4)**: Rb(^t^BuDHP)^[45]^ (0.111 g, 0.5 mmol) was suspended in 3 mL of dry benzene inside a clean dry Schlenk flask and ^i^Bu_2_AlTMP^[43]^ (0.143 g, 0.5 mmol) was added to it. The mixture was stirred for 1 h at room temperature resulting in a pale‐yellow solution. The solvent was evacuated *in vacuo* to form a yellow oil. Upon treating the oil with 2 mL of hexane, crystals crashed out immediately from the oil. Yield=0.188 g, 75 %. A satisfactory elemental analysis for the bulk material of [Rb(^t^BuDHP)(TMP)Al(^i^Bu)_2_] (4) was not obtained, which may be attributed to decomposition during shipping and/or sample preparation. Best values are given, nevertheless. Elemental analysis: Calculated values for [C_26_H_50_AlRbN_2_] (503.14 g mol^−1^): C 62.07, H 10.02, N 5.57; Found: C 61.75, H 9.73, N 4.62. ^1^H NMR [400.03 MHz, 300 K, C_6_D_6_]: *δ* 1.30 ppm (s, 9H, −^t^Bu[DHP]), *δ* 3.85 ppm (d, 1H, H2[DHP]), *δ* 4.56 ppm (dd, 1H, H3[DHP]), *δ* 4.61 ppm (t, 1H, H5[DHP]), *δ* 5.66 ppm (dd, 1H, H4[DHP]), *δ* 7.03 ppm (d, 1H, H6[DHP]), *δ* 0.83 ppm (m, 4H, β‐CH_2_[TMP]), *δ* 1.63 ppm (m, 2H, γCH_2_[TMP]), *δ* 1.34 ppm (s, 6H, −CH_3_[TMP]), *δ* 1.48 ppm (m, 12H, −CH_3_[^i^Bu]+6H, −CH_3_[TMP]), *δ* 2.50 ppm (m, 2H, −CH[^i^Bu]), *δ* 0.43–0.75 ppm (m, 4H, −CH_2_[^i^Bu]); ^13^C {1 H} NMR [C_6_D_6_, 100.60 MHz, 300 K]: *δ* 148.13 ppm (−CH(6)[DHP]), *δ* 125.87 ppm (−CH(4)[DHP]), *δ* 103.95 ppm (−CH(3)[DHP]), *δ* 94.94 ppm (−CH(5)[DHP]), *δ* 60.59 ppm (−CH(2)[DHP]), *δ* 26.30 ppm (−^t^Bu[DHP]), *δ* 41.88 ppm (quaternary[DHP]), *δ* 43.66 ppm (β‐CH_2_[TMP]), *δ* 18.33 ppm (γCH_2_[TMP]), *δ* 33.69+34.08 ppm (CH_3_[TMP]), *δ* 51.55 ppm (quaternary[TMP]), *δ* 27.41+27.75 ppm (−CH[^i^Bu]), *δ* 28.58+29.09+29.68+29.76 ppm (−CH_3_[^i^Bu]), The resonances of −CH_2_[^i^Bu] and quaternary[TMP] could not be observed.

## Conflict of interest

The authors declare no conflict of interest.

## Supporting information

As a service to our authors and readers, this journal provides supporting information supplied by the authors. Such materials are peer reviewed and may be re‐organized for online delivery, but are not copy‐edited or typeset. Technical support issues arising from supporting information (other than missing files) should be addressed to the authors.

Supporting Information
