# Supplementary material for: Hydrocarbon Soluble Alkali‐Metal‐Aluminium Hydride Surrog[ATES]
Source: Chemistry. 2022 Aug 11;28(55):e202201085. doi: 10.1002/chem.202201085 (PMC9804340; doi:10.1002/chem.202201085)
Supplement: Supplementary file 1 — Supporting Information [file CHEM-28-0-s001.pdf]

Chemistry–A European Journal

Supporting Information

Hydrocarbon Soluble Alkali-Metal-Aluminium Hydride Surrog[ATES]

Sumanta Banerjee, Peter A. Macdonald, Samantha A. Orr, Alan R. Kennedy, Alexander van Teijlingen, Stuart D. Robertson,* Tell Tuttle,* and Robert E. Mulvey*

General experimental procedures

All experiments were performed under nitrogen (N_2) atmosphere using standard Schlenk techniques or in a glovebox under argon (Ar) atmosphere. Prior to use, glassware was dried at 150°C and solvents were dried, distilled and degassed using standard methods.[1] n-Hexane, and tetrahydrofuran (THF) were dried by heating to reflux over sodium and benzophenone under N_2. The distilled solvents were collected, degassed, and stored over pre-dried 4 Å molecular sieves before use. n-Pentane and toluene were dried in the Solvent Purification System (Innovative Technology, PS-Micro), degassed, and stored under inert atmosphere over activated 4 Å molecular sieves. Benzene, pyridine, 2,2,6,6-tetramethylpipiridine [TMP(H)], 2-methyl-2-butanol (AmOH) and *N,N,N′,N′*-tetramethylethylenediamine (TMEDA) were obtained from commercial sources and dried over CaH_2 followed by distillation under N_2 atmosphere and were stored over activated 4 Å molecular sieves prior to use. *tert*-Butyllithium (^{t}BuLi), and diisobutylaluminium chloride (^{i}Bu)$_2$AlCl were obtained from commercial sources and used without further purification. 1-Alkali-metal-2-tert-butyl-1,2-dihydropyridide compounds [Li(^{t}BuDHP), Na(^{t}BuDHP), and K(^{t}BuDHP)][2,3] were synthesized and stored in the glove box at -20°C as solid powder following literature procedures. Di-iso-butylaluminium-2,2,6,6-tetramethylpiperidide (^{i}Bu)$_2$Al(TMP)[4] was synthesized according to the literature and stored at room temperature in the glove box as a yellow oil. Rubidium and caesium 1,1,1,3,3,3-hexamethyldisilazide Rb[N(SiMe$_3$)$_2$] and Cs[N(SiMe$_3$)$_2$][5] were synthesized following literature procedure and stored at room temperature in the glove box as white powder. Deuterated solvents [C_6D_6, THF(D8), and C_6D_{12}] were degassed by freeze-pump-thaw method and stored over activated 4 Å molecular sieves. ^{1}H, ^{13}C, ^{7}Li, ^{27}Al, DEPTQ135, COSY, and HSQC NMR spectra were recorded on AV 400 MHz spectrometer. All ^{13}C spectra were proton decoupled. Chemical shifts (δ in ppm) in the ^{1}H and ^{13}C NMR spectra were referenced to the residual signals of the deuterated solvents. ^{7}Li NMR was referenced to 1 M LiCl solution in H_2O (^{7}Li peak set to 0 ppm). For describing signal multiplicities common abbreviations have been used: s (singlet), d (doublet), t (triplet), q (quartet), dd (doublet of a doublet), m (multiplet) and br (broad). Elemental analysis of the crystalline compounds (**1**, **2**, **2a**, **3**, and **4**) were conducted by the Elemental Analysis Service at London Metropolitan University. Single Crystal Diffraction. Crystallographic data for compounds Li(^{t}BuDHP)(TMP)Al(^{i}Bu)$_2$ (**1**), (THF)Li(^{t}BuDHP)(TMP)Al(^{i}Bu)$_2$ (**1a**), [Na(^{t}BuDHP)(TMP)Al(^{i}Bu)$_2$]$_\infty$ (**2**), (TMEDA)Na(^{t}BuDHP)(TMP)Al(^{i}Bu)$_2$ (**2a**), [K(^{t}BuDHP)(TMP)Al(^{i}Bu)$_2$]$_\infty$ (**3**), and [Rb(^{t}BuDHP)(TMP)Al(^{i}Bu)$_2$]$_\infty$ (**4**) were measured with an Oxford Diffraction Gemini E instrument with graphite-monochromated Mo−Kα (λ 0.71073 Å) radiation (**1a** and **2**) or with a Rigaku XtaLAB Synergy-i instrument with monochromated Cu−Kα (λ 1.54184 Å) radiation. The measured data were processed with the CrysAlisPro[6] software package. Using Olex2[7], the structures were solved with the ShelXT[8] structure solution program and refined with ShelXL-2018[9] to convergence and against F^2. Final refinement was within Olex2 or WinGX[10]. All non-hydrogen atoms were refined using anisotropic thermal parameters. Disorder was modelled in a butyl ligand and two THF ligands of **1a** and in a butyl ligand of **2a**. In both cases restraints

and constraints were applied to the disordered groups in order to ensure that their geometries and displacement ellipsoid behaviour approximated normal values. Selected crystallographic and refinement data are given in Table S1.

Openbabel[11] was used to convert the crystallographic information file (CIF) to protein data bank (PDB) files and all calculations were performed using the ORCA 5[12] program.

Synthetic procedures

1. Synthesis of RbOAm

$Rb[N(SiMe_3)_2]$[5] (2.5 g, 10.2 mmol) was transferred into a clean dry Schlenk flask inside the glove box. 10 mL of benzene was added to it using a syringe under inert conditions. To the resultant colourless solution one equivalent of tert-amyl alcohol (AmOH) (1.2 mL, 11 mmol) was added at room temperature and allowed to stir for over an hour. Benzene, and $HN(SiMe_3)_2$ were evacuated under reduced pressure at 60 ^oC to give a white powder which was stored in a vial inside the glove box. Chemical shifts in the ^1H NMR spectrum were identical to that reported in the literature.[13] Yield = 1.501 g, 85 %

2. Synthesis of CsOAm

$Cs[N(SiMe_3)_2]$[5] (3 g, 10.2 mmol) was transferred into a clean dry Schlenk flask inside the glove box. 10 mL of benzene was added to it using a syringe under inert conditions. To the resultant colourless solution one equivalent of tert-amyl alcohol (AmOH) (1.2 mL, 11 mmol) was added at room temperature and allowed to stir for over an hour. Benzene, and $HN(SiMe_3)_2$ were evacuated under reduced pressure at 60 ^oC to give a white powder which was stored in a vial inside the glovebox. Chemical shifts in the ^1H NMR spectrum were identical to those reported in the literature.[13] Yield = 1.975 g, 88 %

3. Synthesis of Rb(^{t}BuDHP)

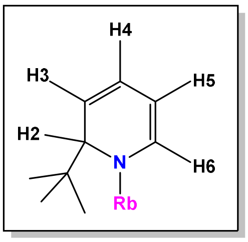

Li(^{t}BuDHP) (0.429 g, 3 mmol) and RbOAm (0.518 g, 3 mmol) were transferred into a clean dry Schlenk flask inside the glove box and 15 mL of benzene was added to it resulting in a pale-yellow suspension which was allowed to stir for 1 hour at room temperature. The solid was then filtered and washed with benzene. The solid was dried *in vacuo* and stored in the glovebox freezer at -20 ^oC. Yield = 0.495 g, 75 %

^1H NMR [400.03 MHz, 300 K, THF(D8)]: δ 0.87 ppm (s, 9H, —^{t}Bu), δ 3.19 ppm (d, 1H, H2), δ 3.65 ppm (dd, 1H, H3), δ 4.22 ppm (dd, 1H, H5), δ 5.81 ppm (dd, 1H, H4), δ 6.71 ppm (d, 1H, H6), $^{13}C\{^1H\}$ NMR [THF(D8), 100.60 MHz, 300 K]: δ 151.4 ppm (—CH(6)), δ 128.5 ppm (—CH(4)), δ 87.8 ppm (—CH(3)), δ 86.3ppm (—CH(5)), δ 68.7 ppm (—CH(2)), δ 37.4 ppm (quaternary[DHP]), δ 25.7 ppm (—^{t}Bu)

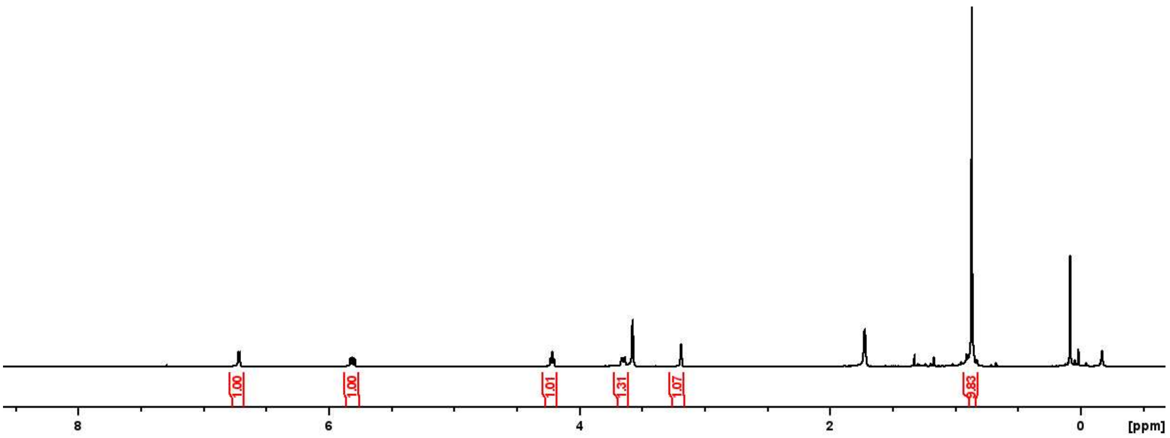

Figure S1: ^{1}H NMR spectrum of Rb(^{t}BuDHP) in THF(D_8)

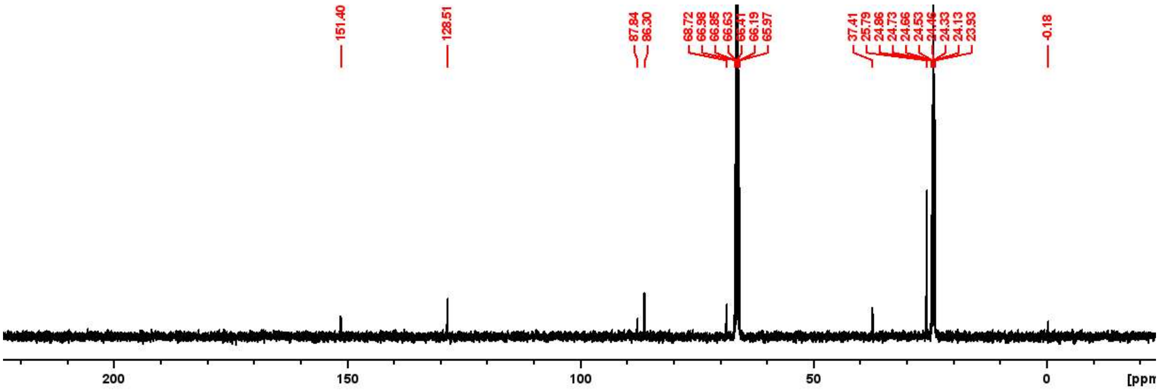

Figure S2: ^{13}C NMR spectrum of Rb(^{t}BuDHP) in THF(D_8)

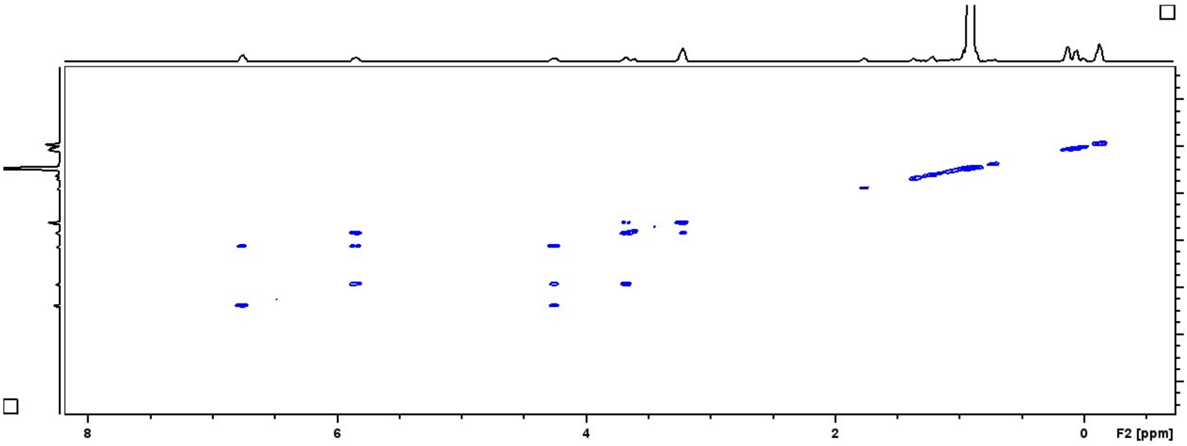

Figure S3: ^{1}H^{1}H-COSY NMR spectrum of Rb(^{t}BuDHP) in THF(D_8)

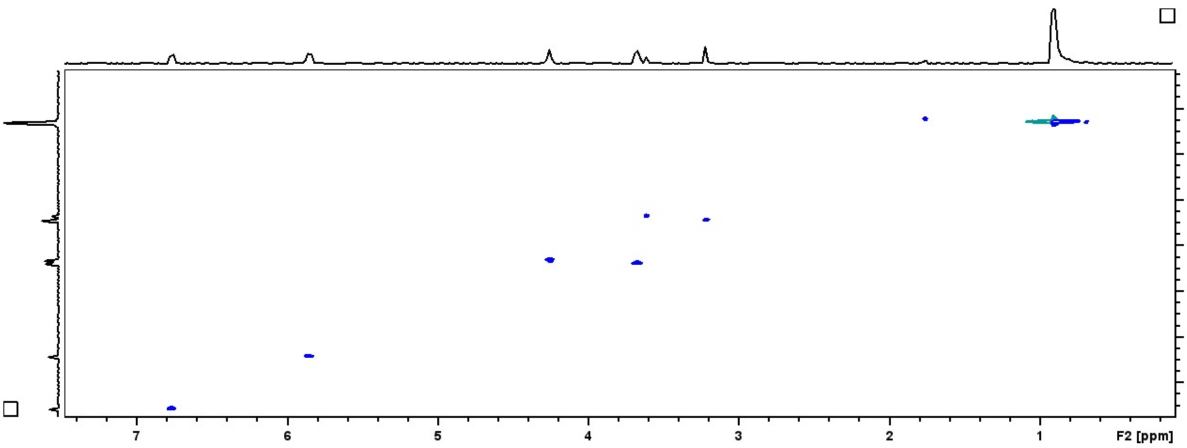

Figure S4: $^{1}H^{13}C$-HSQC NMR spectrum of Rb(^{t}BuDHP) in THF(D_8)

4. Synthesis of Cs(^{t}BuDHP)

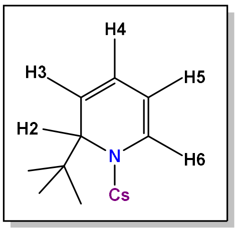

Li(^{t}BuDHP) (0.531 g, 3.72 mmol) and CsOAm (0.820 g, 3.72 mmol) were transferred into a clean dry Schlenk flask inside the glove box and 15 mL of benzene was added to it resulting in a light green suspension which was allowed to stir for 1 hour at room temperature. The solid was then filtered and washed with benzene. The solid was dried *in vacuo* and stored in the glove box freezer at -20 °C. Yield = 0.860 g, 86 %

^{1}H NMR [400.03 MHz, 300 K, THF(D8)]: δ 0.88 ppm (s, 9H, —^{t}Bu), δ 3.31 ppm (d, 1H, H2), δ 3.64 ppm (dd, 1H, H3), δ 4.25 ppm (t, 1H, H5), δ 5.73 ppm (dd, 1H, H4), δ 6.65 ppm (d, 1H, H6), ^{13}C {1 H} NMR [THF(D8), 100.60 MHz, 300 K]: δ 151.3 ppm (—CH(6)), δ 129.2 ppm (—CH(4)), δ 88.3 ppm (—CH(3)), δ 86.3ppm (—CH(5)), δ 68.9 ppm (—CH(2)), δ 37.7 ppm (quaternary[DHP]), δ 26.0 ppm (—^{t}Bu)

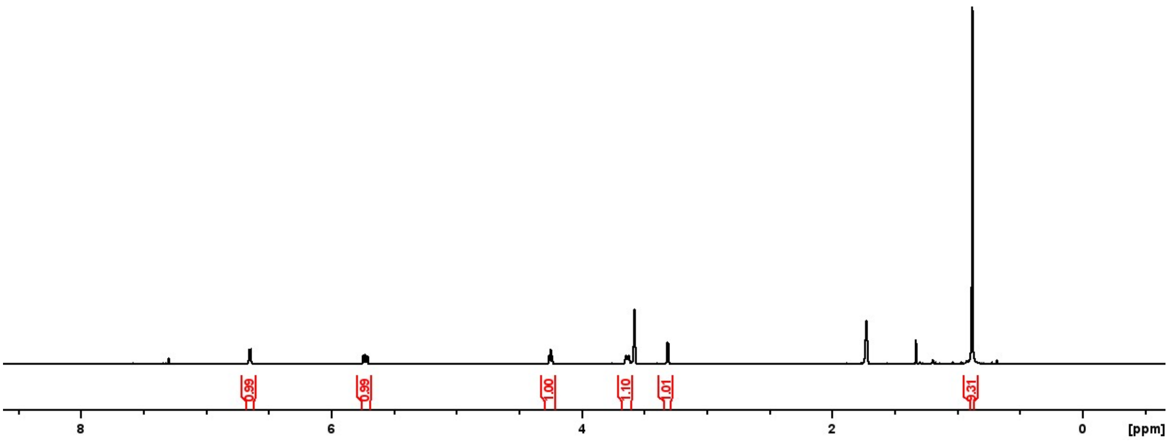

Figure S5: ^{1}H NMR spectrum of Cs(^{t}BuDHP) in THF(D_8)

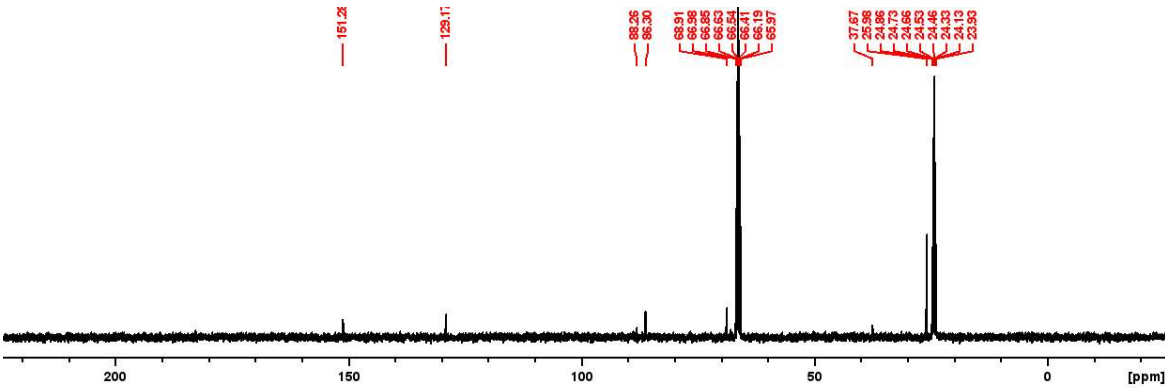

Figure S6: ^{13}C NMR spectrum of Cs(^{t}BuDHP) in THF(D$_8$)

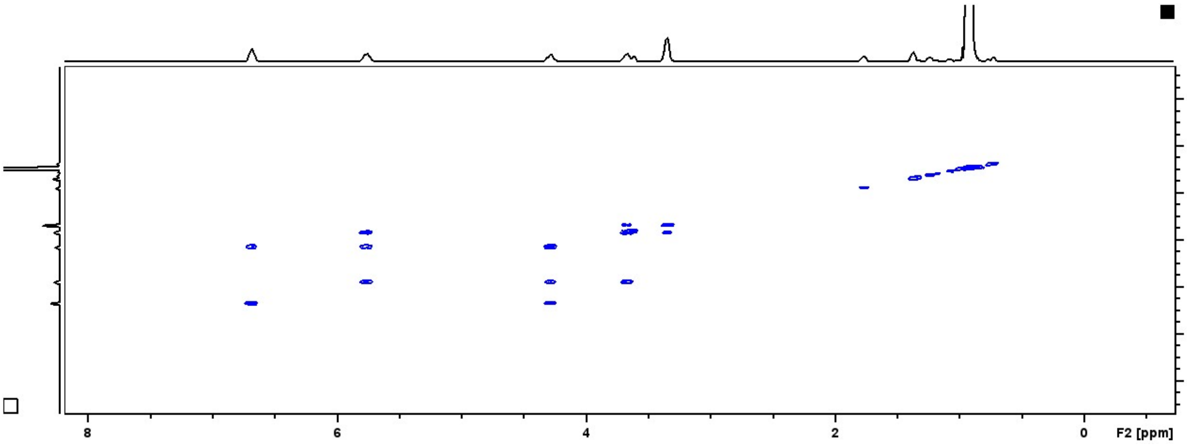

Figure S7: ^{1}H^{1}H-COSY NMR spectrum of Cs(^{t}BuDHP) in THF(D$_8$)

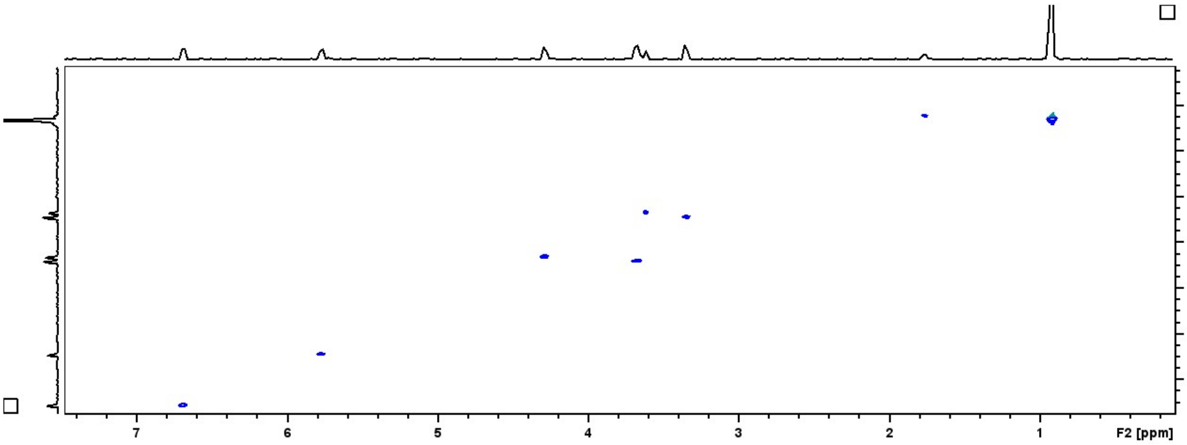

Figure S8: ^{1}H^{13}C-HSQC NMR spectrum of Cs(^{t}BuDHP) in THF(D$_8$)

5. Synthesis of Li(^{t}BuDHP)(TMP)Al(^{i}Bu)$_2$ (1)

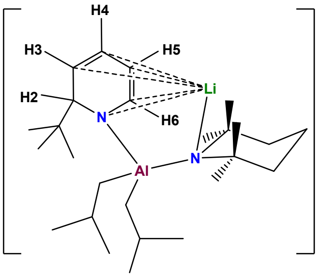

Li(^{t}BuDHP) (0.143 g, 1 mmol) was suspended in 3 mL of dry n-pentane inside a clean dry Schlenk flask and ^{i}Bu$_2$AlTMP (0.281 g, 1 mmol) was added to it. The mixture was stirred for 1 hour at room temperature resulting in a pale-yellow solution. Upon concentrating and storing the solution at -20 ^{o}C colourless blocks of crystals were obtained overnight. Yield = 0.298 g, 70 %

Elemental analysis: Calculated values for $C_{26}H_{50}AlLiN_2$ (424.61 g/mol): C 73.54, H 11.87, N 6.60; Found: C 73.50, H 11.82, N 6.43.

^{1}H NMR [400.03 MHz, 300 K, C_6D_{12}]: δ 0.98 ppm (s, 9H, —^{t}Bu[DHP]), δ 3.83 ppm (d, 1H, H2[DHP]), δ 4.94 ppm (dd, 1H, H3[DHP]), δ 5.34 ppm (dd, 1H, H5[DHP]), δ 6.27 ppm (dd, 1H, H4[DHP]), δ 7.14 ppm (d, 1H, H6[DHP]), δ 1.72 ppm (m, 2H, β-CH_2[TMP]), δ 0.92 ppm (m, 1H, β-CH_2[TMP]), δ 0.80 ppm (m, 1H, β-CH_2[TMP]), δ 1.96 ppm (m, 1H, γCH_2[TMP]), δ 1.47-1.54 ppm (m, 12H, —CH_3[TMP] + 1H, γCH_2[TMP]), δ 1.11-1.16 ppm (m, 12H, —CH_3[^{i}Bu]), δ 2.13 ppm (m, 2H, —CH[^{i}Bu]), δ 0.19-0.35 ppm (m, 2H, —CH_2[^{i}Bu]), δ 0.49-0.57 ppm (m, 2H, —CH_2[^{i}Bu]); ^{13}C {1 H} NMR [C_6D_{12}, 100.60 MHz, 300 K]: δ 147.62 ppm (—CH(6)[DHP]), δ 123.42 ppm (—CH(4)[DHP]), δ 107.16 ppm (—CH(3)[DHP]), δ 99.09 ppm (—CH(5)[DHP]), δ 59.70 ppm (—CH(2)[DHP]), δ 24.76 ppm (—^{t}Bu[DHP]), δ 40.68 ppm (quaternary[DHP]), δ 45.45 + 46.24 ppm (β-CH_2[TMP]), δ 17.81 ppm (γCH_2[TMP]), δ 36.69 + 38.01 ppm (CH_3[TMP]), δ 51.99 + 51.76 ppm (2 × quaternary[TMP]), δ 26.73 ppm (—CH[^{i}Bu]), δ 27.97 + 28.10 ppm (—CH_3[^{i}Bu]), The resonances of —CH_2[^{i}Bu] could not be observed.[14]

^{7}Li (C_6D_{12}, 155.50 MHz, 300 K) δ = -1.71 ppm (s).

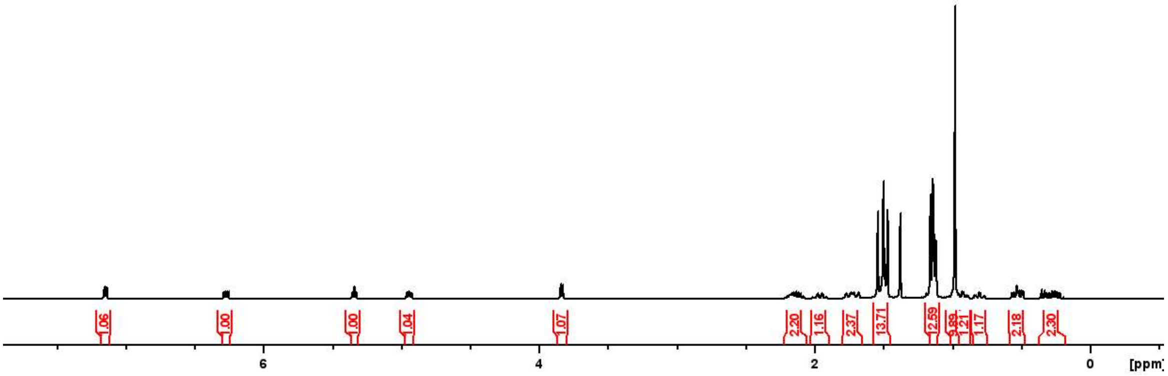

Figure S9: ^{1}H NMR spectrum of Li(^{t}BuDHP)(TMP)Al(^{i}Bu)$_2$ **(1)** in C_6D_{12}

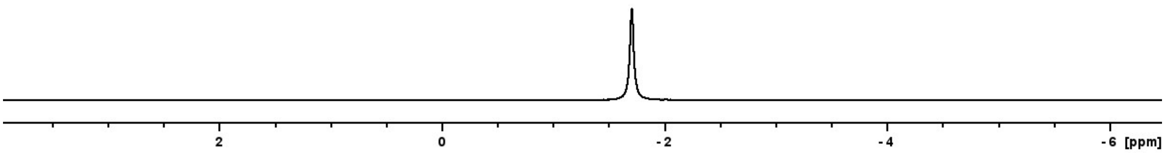

Figure S10: ^{7}Li NMR spectrum of Li(^{t}BuDHP)(TMP)Al(^{i}Bu)$_2$ **(1)** in C_6D_{12}

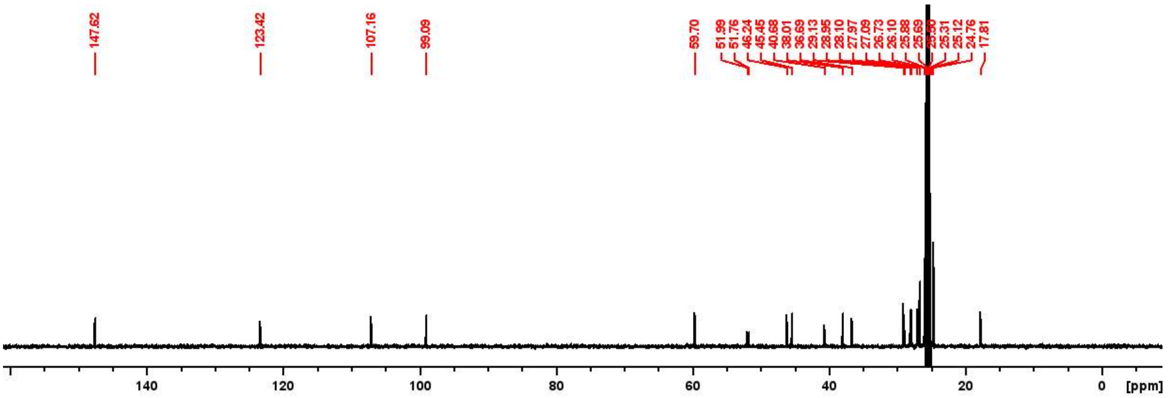

Figure S11: ^{13}C NMR spectrum of Li(^{t}BuDHP)(TMP)Al(^{i}Bu)$_2$ **(1)** in C_6D_{12}

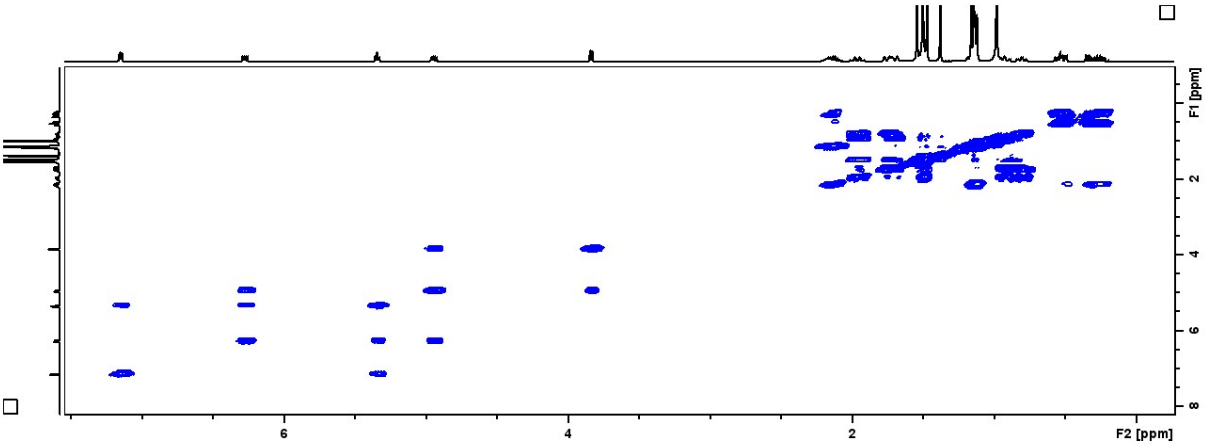

Figure S12: ^{1}H^{1}H-COSY NMR spectrum of Li(^{t}BuDHP)(TMP)Al(^{i}Bu)$_2$ **(1)** in C_6D_{12}

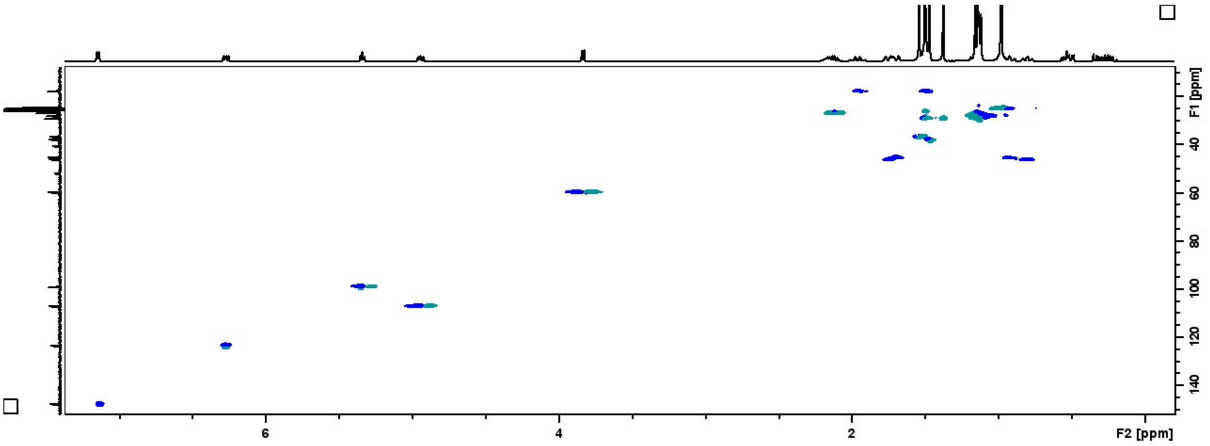

Figure S13: ^{1}H^{13}C-HSQC NMR spectrum of Li(^{t}BuDHP)(TMP)Al(^{i}Bu)$_2$ **(1)** in C_6D_{12}

6. Synthesis of [(THF)Li(^{t}BuDHP)(TMP)Al(^{i}Bu)$_2$)] (1a)

Li(^{t}BuDHP) (0.143 g, 1 mmol) and ^{i}Bu$_2$AlTMP (0.281 g, 1 mmol) were added to a clean dry Schlenk flask along with 10 mL of n-hexane at room temperature. This was left to stir for 10 minutes. To obtain a completely soluble solution, THF was added dropwise. This resulted in a colourless oil forming, from which crystals grew from at -30 °C. Yield = 0.078 g, 16 % Due to the low yield, clean NMR spectra and Elemental Analysis could not be obtained.

7. Synthesis of [Na(^{t}BuDHP)(TMP)Al(^{i}Bu)]$_\infty$ (2)

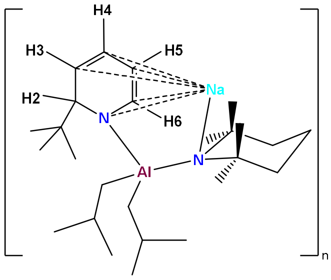

Na(^{t}BuDHP) (0.159 g, 1 mmol) was suspended in 3 mL of dry benzene inside a clean dry Schlenk flask and $^{i}Bu_2AlTMP$ (0.281 g, 1 mmol) was added to it. The mixture was stirred for 1 hour at room temperature resulting in a yellow solution. Upon layering the concentrated benzene solution with n-hexane at room temperature, colourless blocks of crystals were obtained in two days. Yield = 0.295 g, 67 %

Elemental analysis: Calculated values for [$C_{26}H_{50}AlNaN_2$] (440.66 g/ mol): C 70.87, H 11.44, N 6.36; Found: C 70.80, H 11.36, N 5.98.

^{1}H NMR [400.03 MHz, 300 K, C_6D_6]: δ 1.18 ppm (s, 9H, —^{t}Bu[DHP]), δ 3.76 ppm (d, 1H, H2[DHP]), δ 4.59 ppm (dd, 1H, H3[DHP]), δ 4.74 ppm (t, 1H, H5[DHP]), δ 5.85 ppm (dd, 1H, H4[DHP]), δ 7.11 ppm (d, 1H, H6[DHP]), δ 1.35 ppm (m, 2H, β-CH_2[TMP]), δ -0.30 ppm (t, 1H, β-CH_2[TMP]), δ -0.02 ppm (t, 1H, β-CH_2[TMP]), δ 1.73 ppm (m, 1H, γCH_2[TMP]), δ 1.09 + 1.15 + 1.39 + 1.51 ppm (br s, 4 × 3H, —CH_3[TMP]), δ 1.09 ppm (br s, 1H, γCH_2[TMP]), δ 1.43-1.47 ppm (m, 12H, —CH_3[^{i}Bu]), δ 2.46 ppm (m, 2H, —CH[^{i}Bu]), δ 0.38-0.87 ppm (m, 4H, —CH_2[^{i}Bu]); ^{13}C {1 H} NMR [C_6D_6, 100.60 MHz, 300 K]: δ 147.65 ppm (—CH(6)[DHP]), δ 125.41 ppm (—CH(4)[DHP]), δ 105.02 ppm (—CH(3)[DHP]), δ 94.96 ppm (—CH(5)[DHP]), δ 59.96 ppm (—CH(2)[DHP]), δ 25.66 ppm (—^{t}Bu[DHP]), δ 41.55 ppm (quaternary[DHP]), δ 45.27 ppm (β-CH_2[TMP]), δ 17.72 ppm (γCH_2[TMP]), δ 37.56 + 38.80 ppm (CH_3[TMP]), δ 27.34 + 27.51 ppm (—CH[^{i}Bu]), δ 28.24 + 28.59 + 29.27 + 29.94 ppm (—CH_3[^{i}Bu]), The resonances of —CH_2[^{i}Bu] and quaternary[TMP] could not be observed.

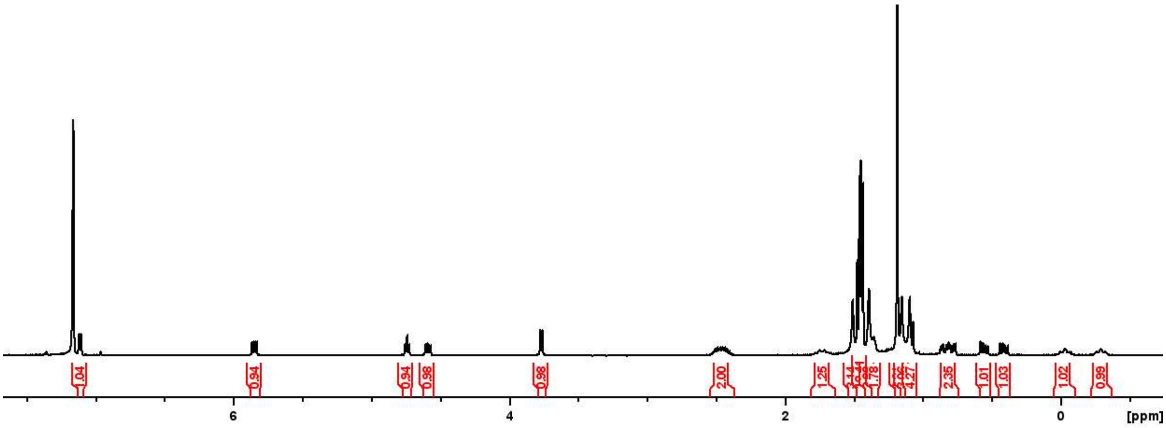

Figure S14: ^{1}H NMR spectrum of [Na(^{t}BuDHP)(TMP)Al(^{i}Bu)$_2$]$_n$ **(2)** in C_6D_6

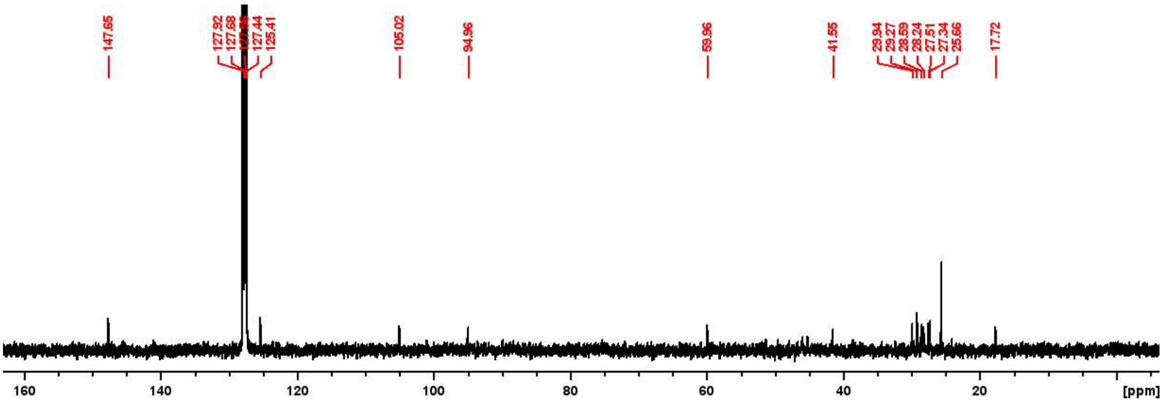

Figure S15: ^{13}C NMR spectrum of [Na(^{t}BuDHP)(TMP)Al(^{i}Bu)$_2$]$_n$ **(2)** in C_6D_6

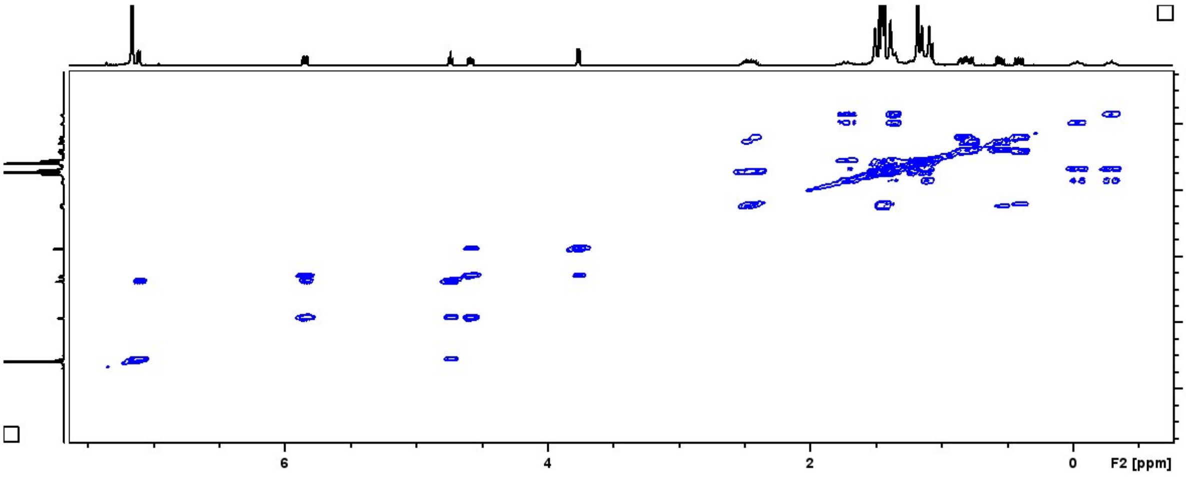

Figure S16: ^{1}H^{1}H-COSY NMR spectrum of [Na(^{t}BuDHP)(TMP)Al(^{i}Bu)$_2$]$_n$ **(2)** in C_6D_6

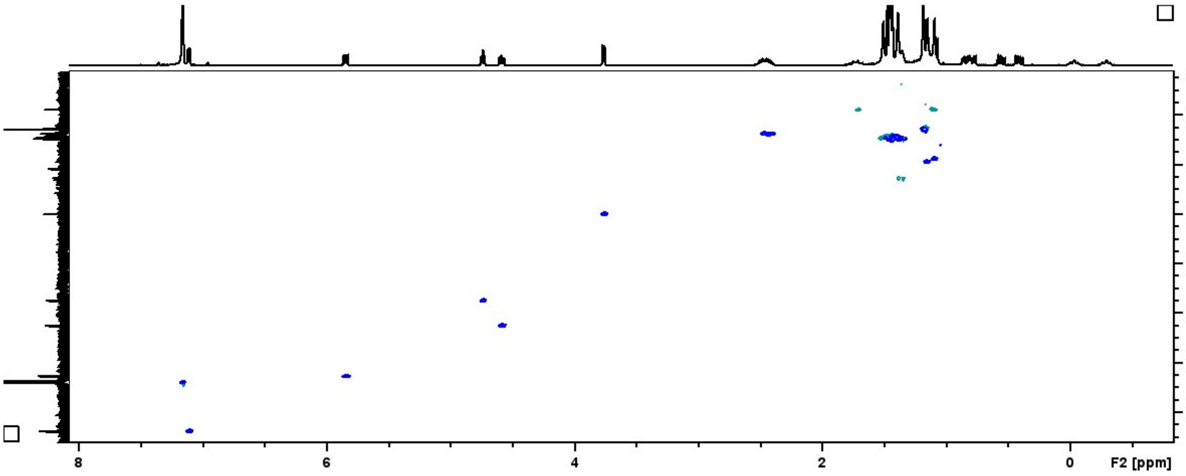

Figure S17: ^{1}H^{13}C-HSQC NMR spectrum of [Na(^{t}BuDHP)(TMP)Al(^{i}Bu)$_2$]$_n$ **(2)** in C_6D_6

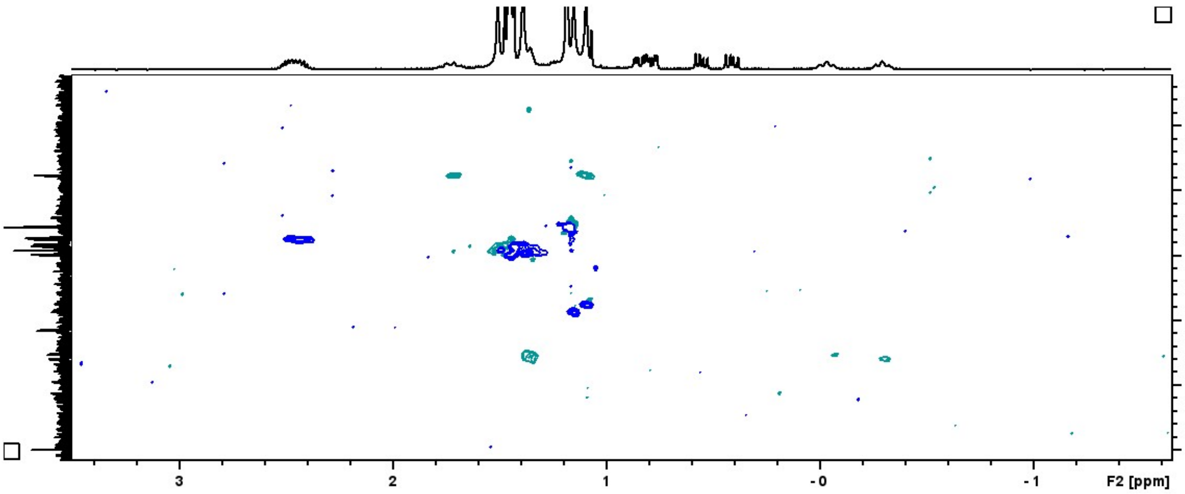

Figure S18: $^{1}H^{13}C$-HSQC NMR spectrum (expanded) of [Na(^{t}BuDHP)(TMP)Al(^{i}Bu)$_2$]$_n$ **(2)** in C_6D_6 depicting the γ-TMP and β-TMP ^{13}C—^{1}H correlation.

8. Synthesis of [(TMEDA)Na(^{t}BuDHP)(TMP)Al(^{i}Bu)] (2a)

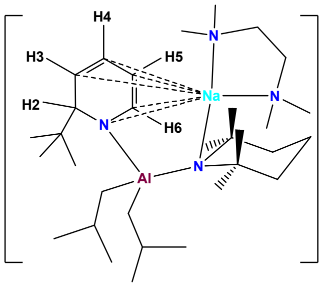

Na(^{t}BuDHP) (0.018 g, 0.11 mmol) was suspended in 0.5 mL of dry benzene inside a clean dry vial in the glove box and ^{i}Bu$_2$AlTMP (0.033 g, 0.11 mmol) was added to it. The mixture was stirred for 30 minutes at room temperature resulting in a yellow solution. An equivalent amount of TMEDA (16 µL, 0.11 mmol) was added dropwise and the resultant mixture was layered with n-pentane. Colourless blocks of crystals were obtained after thirty days at - 20°C. Yield = 0.038 g, 63 %

A satisfactory elemental analysis for the bulk material of [(TMEDA)Na(^{t}BuDHP)(TMP)Al(^{i}Bu)$_2$] (**2a**) was not obtained, which may be attributed to decomposition during shipping and/or sample preparation. Best values are given, nevertheless. Elemental analysis: Calculated values for $C_{32}H_{66}AlN_4Na$ (556.86 g/ mol): C 69.02, H 11.95, N 10.06; Found: C 68.98, H 11.73, N 7.59.

^{1}H NMR [400.03 MHz, 300 K, C_6D_{12}]: δ 0.94 ppm (s, 9H, —^{t}Bu[DHP]), δ 3.74 ppm (d, 1H, H2[DHP]), δ 4.70 ppm (dd, 1H, H3[DHP]), δ 4.76 ppm (t, 1H, H5[DHP]), δ 6.02 ppm (dd, 1H, H4[DHP]), δ 7.04 ppm (d, 1H, H6[DHP]), δ 2.37 ppm (s, 4H, —CH$_2$[TMEDA]), δ 2.27 ppm (s, 12H, —CH$_3$[TMEDA]), δ 1.70 ppm (br s, 2H, γCH$_2$[TMP]), δ 1.31-1.41 ppm (m, 12H, —CH$_3$[TMP] + 4H, β-CH$_2$[TMP]), δ 1.04 ppm (m, 12H, —CH$_3$[^{i}Bu]), δ 2.06 ppm (m, 2H, —CH[^{i}Bu]), δ 0.04-0.47 ppm (m, 4H, —CH$_2$[^{i}Bu]); ^{13}C {1 H} NMR [C_6D_{12}, 100.60 MHz, 300 K]: δ 147.06 ppm (—CH(6)[DHP]), δ 123.98 ppm (—CH(4)[DHP]), δ 105.62 ppm (—CH(3)[DHP]), δ 93.75 ppm (—CH(5)[DHP]), δ 60.21 ppm (—CH(2)[DHP]), δ 25.19 ppm (—(CH$_3$)$_3$[DHP]), δ 41.32 ppm (quaternary[DHP]), δ 57.91 ppm (—CH$_2$[TMEDA]), δ 46.73 ppm (—CH$_3$[TMEDA]), δ 45.83 ppm (β-CH$_2$[TMP]), δ 18.16 ppm (γCH$_2$[TMP]), δ 26.09 ppm (CH$_3$[TMP]), δ 51.65 ppm (quaternary[TMP]), δ 26.92 + 27.03 ppm (—CH[^{i}Bu]), δ 27.97 + 28.60 + 29.06 ppm (—CH$_3$[^{i}Bu]), The resonances of —CH$_2$[^{i}Bu] could not be observed.

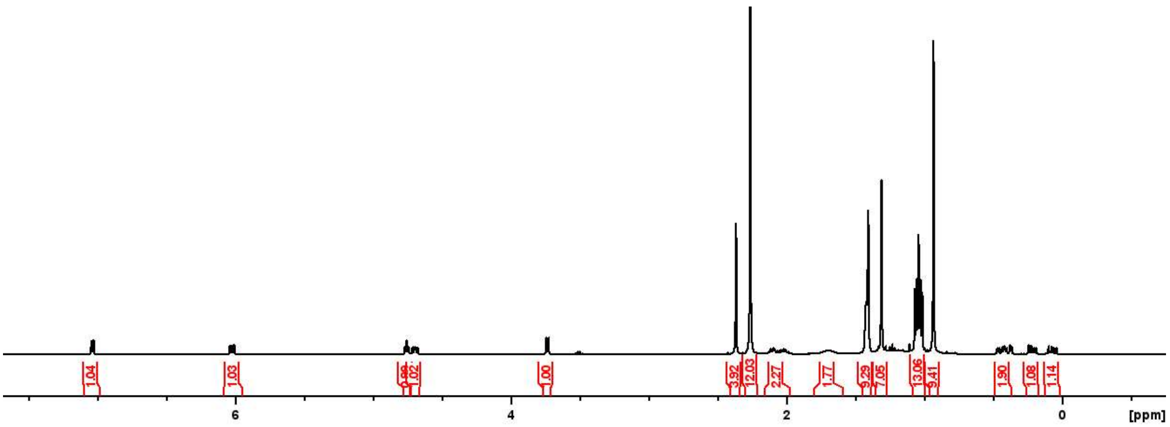

Figure S19: ^{1}H NMR spectrum of [(TMEDA)Na(^{t}BuDHP)(TMP)Al(^{i}Bu)$_2$] **(2a)** in C_6D_{12}

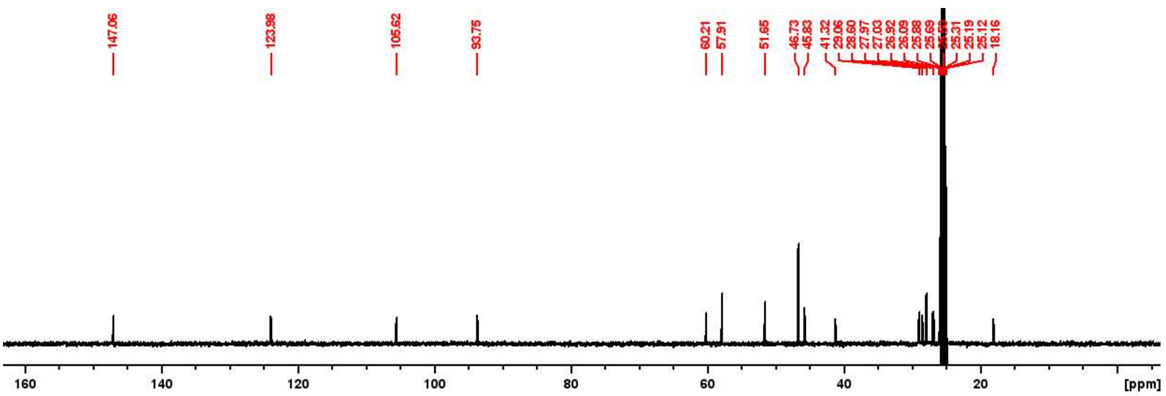

Figure S20: ^{13}C NMR spectrum of [(TMEDA)Na(^{t}BuDHP)(TMP)Al(^{i}Bu)$_2$] **(2a)** in C_6D_{12}

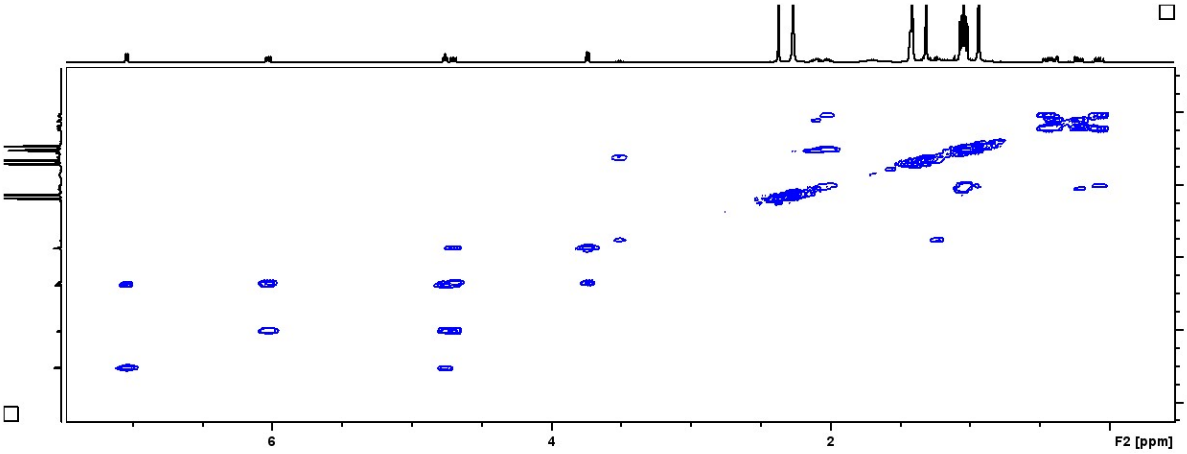

Figure S21: ^{1}H^{1}H-COSY NMR spectrum of [(TMEDA)Na(^{t}BuDHP)(TMP)Al(^{i}Bu)$_2$] **(2a)** in C_6D_{12}

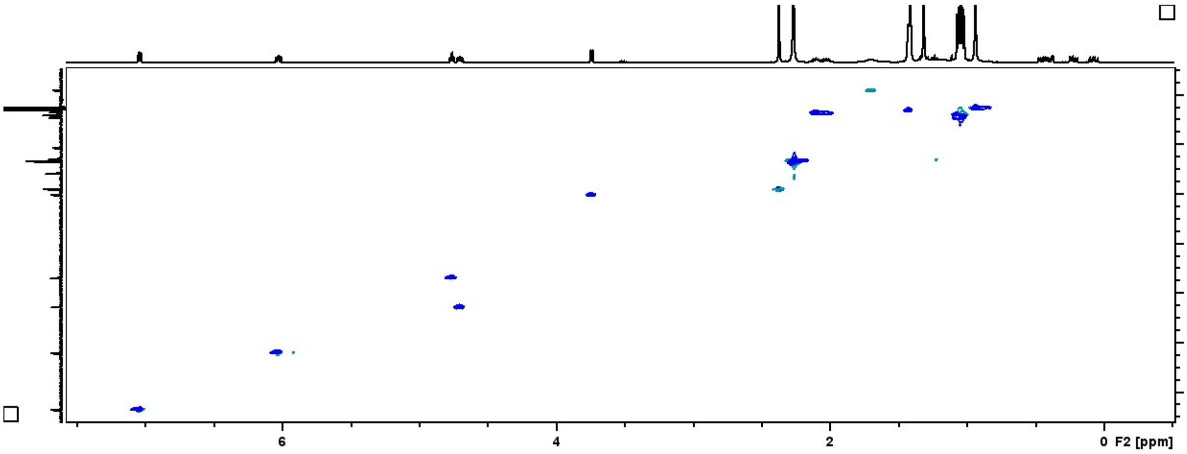

Figure S22: $^{1}H^{13}C$-HSQC NMR spectrum of [(TMEDA)Na(^{t}BuDHP)(TMP)Al(^{i}Bu)$_2$] **(2a)** in C_6D_{12}

9. Synthesis of [K(^{t}BuDHP)(TMP)Al(^{i}Bu)]$_\infty$ (3)

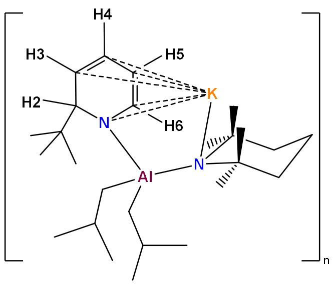

K(^{t}BuDHP) (0.129 g, 0.75 mmol) was suspended in 3 mL of dry benzene inside a clean dry Schlenk flask and $^{i}Bu_2$AlTMP (0.215 g, 0.75 mmol) was added to it. The mixture was stirred for 1 hour at room temperature. resulting in an orange solution. The solvent was evacuated *in vacuo* to form a yellow oil. Upon treating the oil with 2 mL of hexane crystals crashed out immediately from the oil. Yield = 0.253 g, 74 %

A satisfactory elemental analysis for the bulk material of [K(^{t}BuDHP)(TMP)Al(^{i}Bu)$_2$] (**3**) was not obtained, which may be attributed to decomposition during shipping and/or sample preparation. Best values are given, nevertheless. Elemental analysis: Calculated values for [$C_{26}H_{50}AlKN_2$] (456.77 g/ mol): C 68.37, H 11.03, N 6.13; Found: C 67.84, H 9.98, N 4.67.

^{1}H NMR [400.03 MHz, 300 K, C_6D_6]: δ 1.25 ppm (br s, 9H, —(CH_3)$_3$[DHP] + 6H, —CH_3[TMP] + 2H, β-CH_2[TMP]), δ 3.79 ppm (d, 1H, H2[DHP]), δ 4.54 ppm (t, 1H, H3[DHP]), δ 4.67 ppm (dd, 1H, H5[DHP]), δ 5.74 ppm (dd, 1H, H4[DHP]), δ 7.04 ppm (d, 1H, H6[DHP]), δ 1.38 ppm (m, 6H, —CH_3[TMP]), δ 0.87 ppm (m, 1H, β-CH_2[TMP]), δ 1.47 ppm (m, 12H, —CH_3[^{i}Bu] + 2H, γCH_2[TMP] + 1H, β-CH_2[TMP]), δ 2.50 ppm (m, 2H, —CH[^{i}Bu]), δ 0.41-0.83 ppm (m, 4H, —CH_2[^{i}Bu]); ^{13}C {1 H} NMR [C_6D_6, 100.60 MHz, 300 K]: δ 147.92 ppm (—CH(6)[DHP]), δ 125.67 ppm (—CH(4)[DHP]), δ 104.02 ppm (—CH(3)[DHP]), δ 95.18 ppm (—CH(5)[DHP]), δ 66.28 ppm (—CH(2)[DHP]), δ 26.08 ppm (—^{t}Bu[DHP]), δ 41.78 ppm (quaternary[DHP]), δ 18.13 ppm (γCH_2[TMP]), δ 51.50 ppm (quaternary[TMP]), δ 27.44 + 27.73 ppm (—CH[^{i}Bu]), δ 28.44 + 28.76 + 29.63 + 29.88 ppm (—CH_3[^{i}Bu]). The resonances of —CH_2[^{i}Bu] could not be observed. The resonances of β-CH_2[TMP] and —CH_3[TMP] were not assigned due to overlap of the methyl signals (from TMP and ^{i}Bu groups) in $^{1}H^{13}C$-HSQC NMR spectrum.

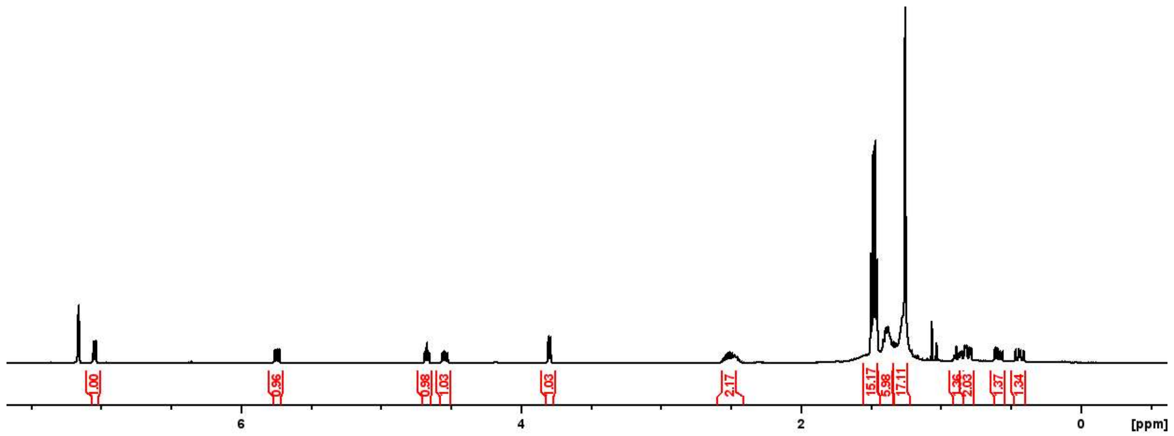

Figure S23: ^{1}H NMR spectrum of $[K(^{t}BuDHP)(TMP)Al(^{i}Bu)_2]_n$ **(3)** in C_6D_6

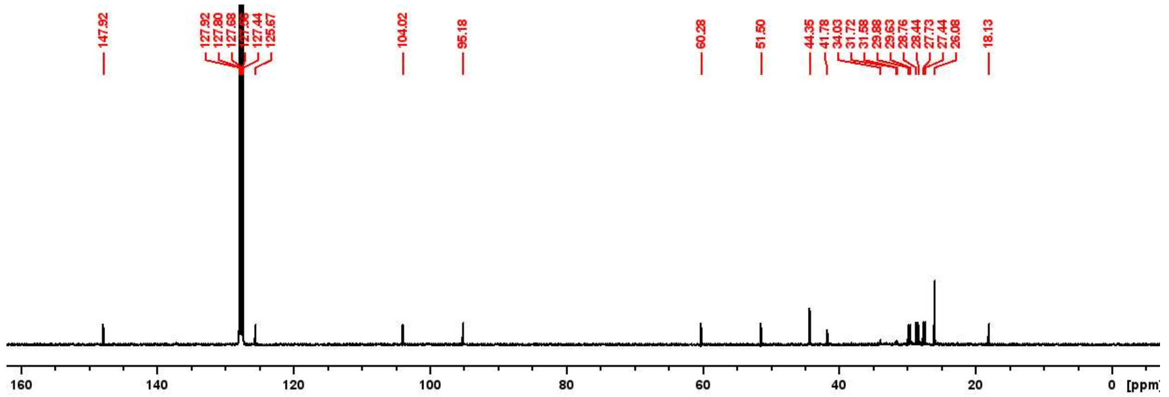

Figure S24: ^{13}C NMR spectrum of $[K(^{t}BuDHP)(TMP)Al(^{i}Bu)_2]_n$ **(3)** in C_6D_6

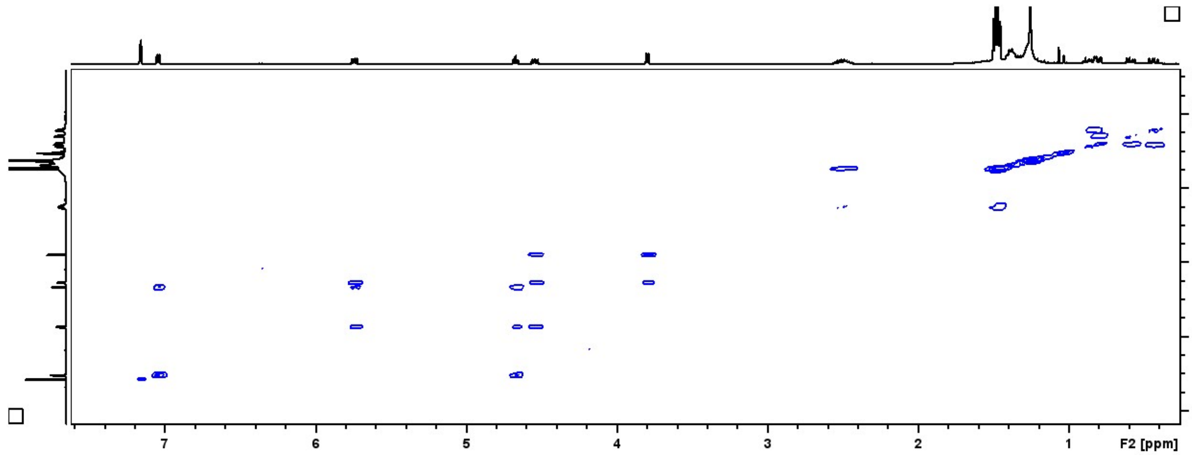

Figure S25: $^{1}H^{1}H$-COSY NMR spectrum of $[K(^{t}BuDHP)(TMP)Al(^{i}Bu)_2]_n$ **(3)** in C_6D_6

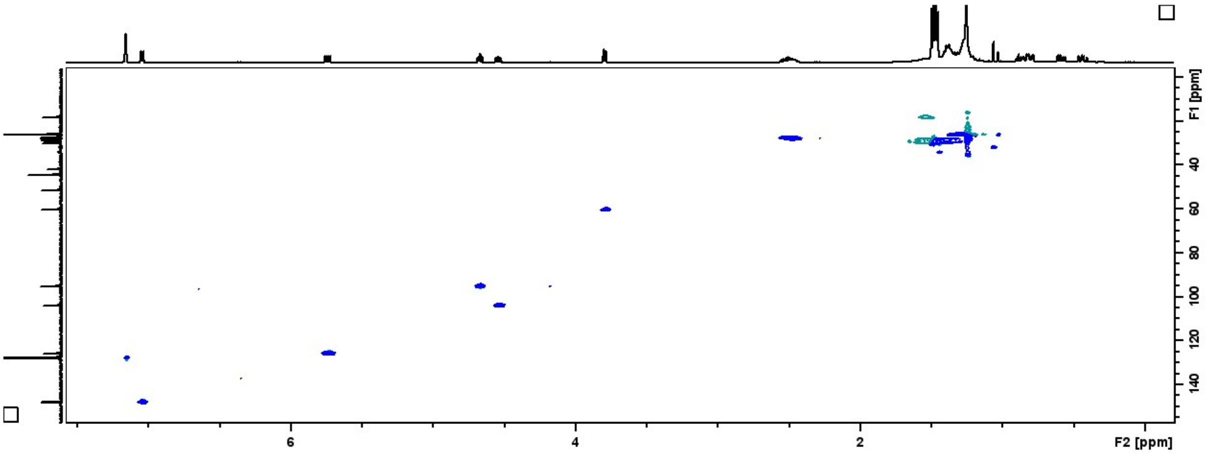

Figure S26: $^{1}H^{13}C$-HSQC NMR spectrum of [K(^{t}BuDHP)(TMP)Al(^{i}Bu)$_2$]$_n$ **(3)** in C_6D_6

10. Synthesis of [Rb(DHP)(TMP)Al(^{i}Bu)]$_\infty$ (4)

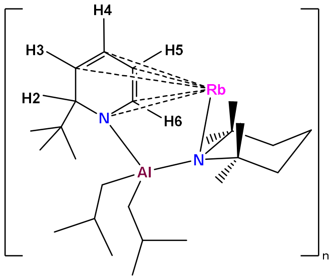

Rb(^{t}BuDHP) (0.111 g, 0.5 mmol) was suspended in 3 mL of dry benzene inside a clean dry Schlenk flask and $^{i}Bu_2$AlTMP (0.143 g, 0.5 mmol) was added to it. The mixture was stirred for 1 hour at room temperature. resulting in a pale-yellow solution. The solvent was evacuated *in vacuo* to form a yellow oil. Upon treating the oil with 2 mL of hexane, crystals crashed out immediately from the oil. Yield = 0.188 g, 75 %

A satisfactory elemental analysis for the bulk material of [Rb(^{t}BuDHP)(TMP)Al(^{i}Bu)$_2$] (**4**) was not obtained, which may be attributed to decomposition during shipping and/or sample preparation. Best values are given, nevertheless. Elemental analysis: Calculated values for [$C_{26}H_{50}AlRbN_2$] (503.14 g/ mol): C 62.07, H 10.02, N 5.57; Found: C 61.75, H 9.73, N 4.62.

^{1}H NMR [400.03 MHz, 300 K, C_6D_6]: δ 1.30 ppm (s, 9H, —^{t}Bu[DHP]), δ 3.85 ppm (d, 1H, H2[DHP]), δ 4.56 pp (dd, 1H, H3[DHP]), δ 4.61 ppm (t, 1H, H5[DHP]), δ 5.66 ppm (dd, 1H, H4[DHP]), δ 7.03 ppm (d, 1H, H6[DHP]), δ 0.83 ppm (m, 4H, β-CH_2[TMP]), δ 1.63 ppm (m, 2H, γCH_2[TMP]), δ 1.34 ppm (s, 6H, —CH_3[TMP]), δ 1.48 ppm (m, 12H, —CH_3[^{i}Bu] + 6H, —CH_3[TMP]), δ 2.50 ppm (m, 2H, —CH[^{i}Bu]), δ 0.43-0.75 ppm (m, 4H, —CH_2[^{i}Bu]); ^{13}C {1 H} NMR [C_6D_6, 100.60 MHz, 300 K]: δ 148.13 ppm (—CH(6)[DHP]), δ 125.87 ppm (—CH(4)[DHP]), δ 103.95 ppm (—CH(3)[DHP]), δ 94.94 ppm (—CH(5)[DHP]), δ 60.59 ppm (—CH(2)[DHP]), δ 26.30 ppm (—^{t}Bu[DHP]), δ 41.88 ppm (quaternary[DHP]), δ 43.66 ppm (β-CH_2[TMP]), δ 18.33 ppm (γCH_2[TMP]), δ 33.69 + 34.08 ppm (CH_3[TMP]), δ 51.55 ppm (quaternary[TMP]), δ 27.41 + 27.75 ppm (—CH[^{i}Bu]), δ 28.58 + 29.09 + 29.68 + 29.76 ppm (—CH_3[^{i}Bu]), The resonances of —CH_2[^{i}Bu] and quaternary[TMP] could not be observed.

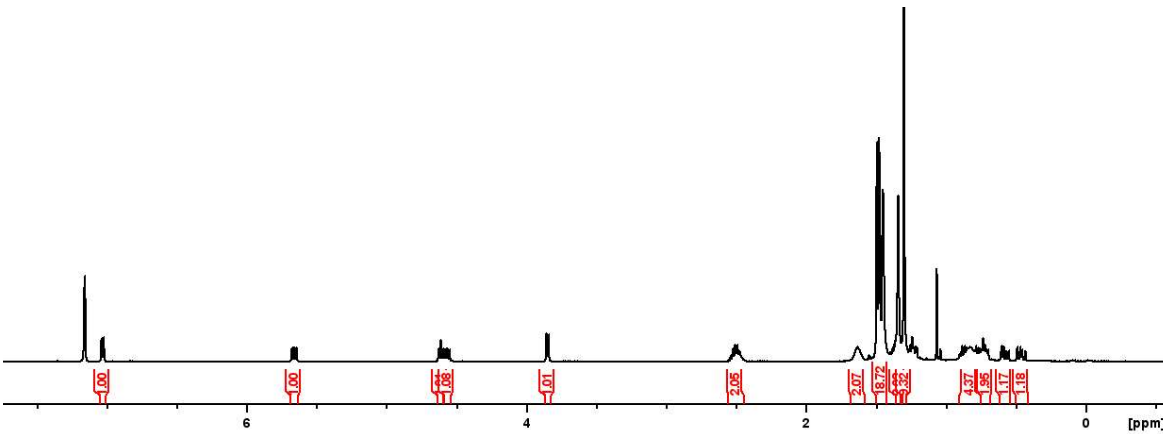

Figure S27: ^{1}H NMR spectrum of $[Rb(^{t}BuDHP)(TMP)Al(^{i}Bu)_2]_n$ **(4)** in C_6D_6

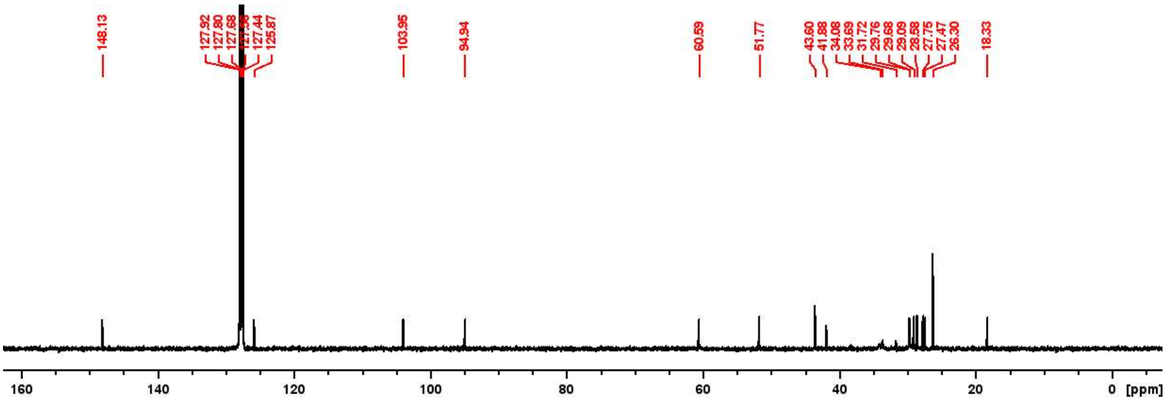

Figure S28: ^{13}C NMR spectrum of $[Rb(^{t}BuDHP)(TMP)Al(^{i}Bu)_2]_n$ **(4)** in C_6D_6

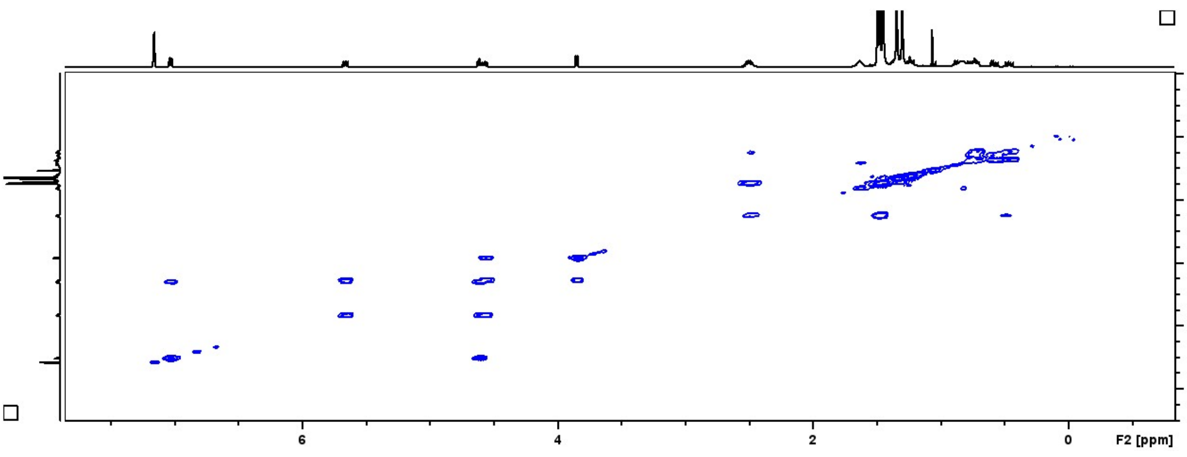

Figure S29: ^{1}H^{1}H-COSY NMR spectrum of $[Rb(^{t}BuDHP)(TMP)Al(^{i}Bu)_2]_n$ **(4)** in C_6D_6

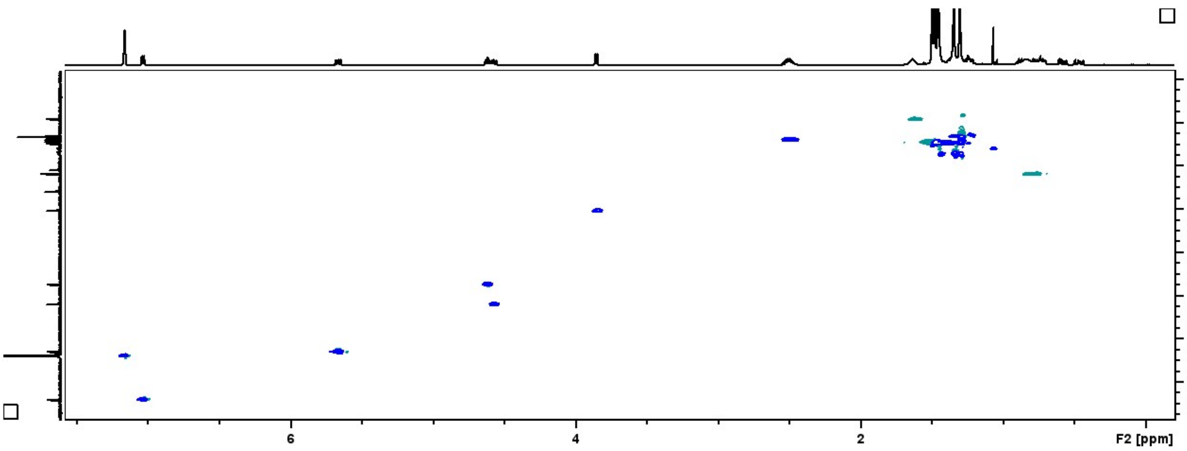

Figure S30: $^{1}H^{13}C$-HSQC NMR spectrum of [Rb(^{t}BuDHP)(TMP)Al(^{i}Bu)$_2$]$_n$ **(4)** in C_6D_6

11. NMR reaction of Cs(^{t}BuDHP) + $^{i}Bu_2$AlTMP in C_6D_6

This reaction was carried out in an identical fashion to that employed to prepare the Rb derivative **4**, above. Unfortunately, no solid product could be obtained and purified and so this was carried out on an NMR scale to obtain spectra for comparison.

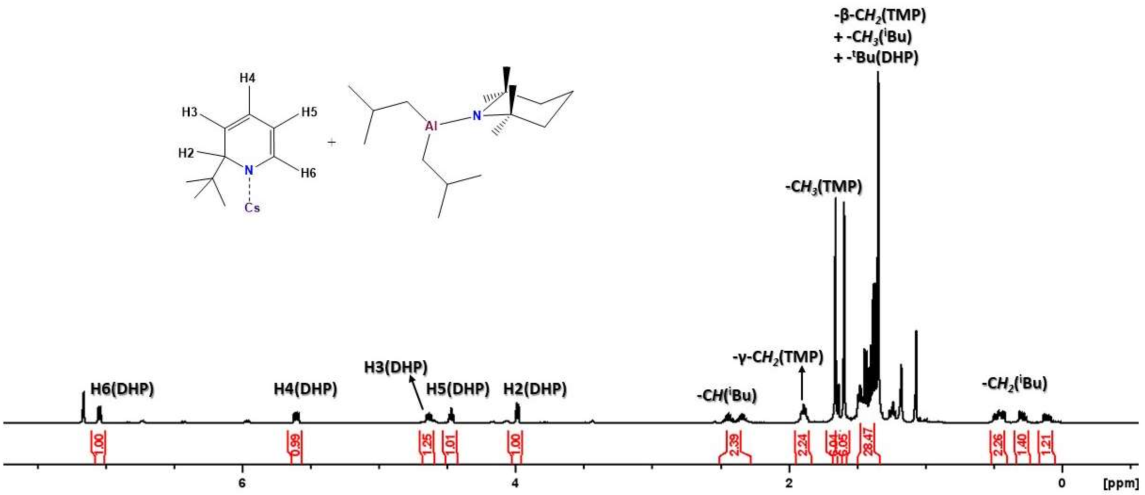

Figure S31: ^{1}H NMR spectrum of [Cs(^{t}BuDHP)] + [(TMP)Al(^{i}Bu)$_2$] in C_6D_6

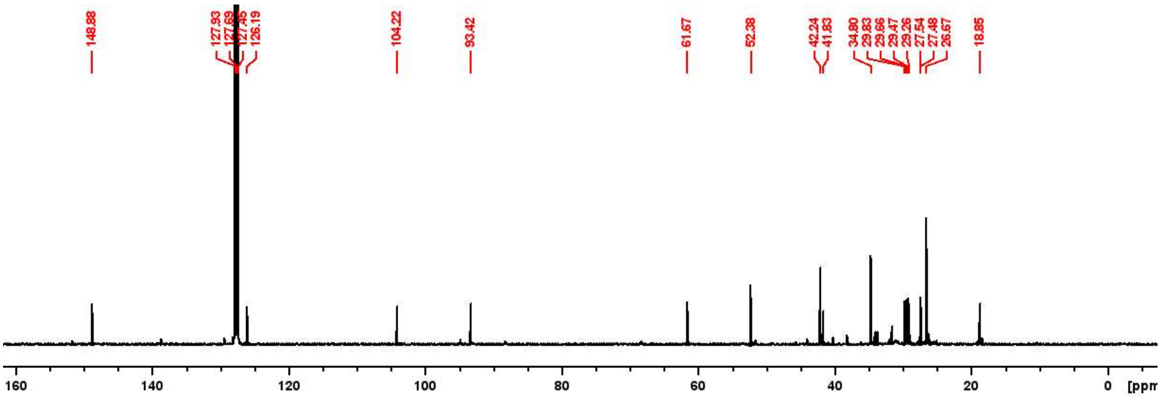

Figure S32: ^{13}C NMR spectrum of [Cs(^{t}BuDHP)] + [(TMP)Al(^{i}Bu)$_2$] in C_6D_6

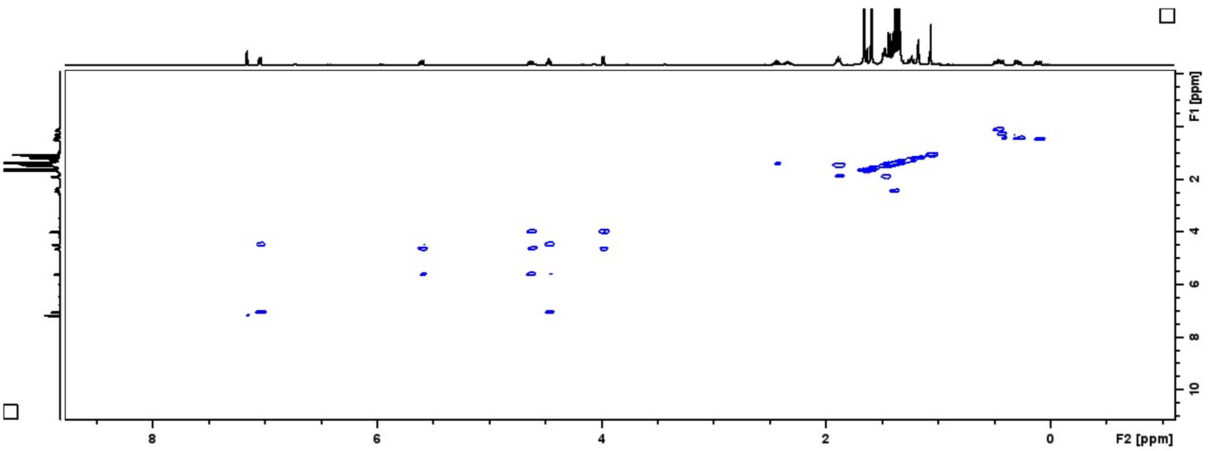

Figure S33: $^1H^1H$-COSY NMR spectrum of [Cs(^{t}BuDHP)] + [(TMP)Al(^{i}Bu)$_2$] in C_6D_6

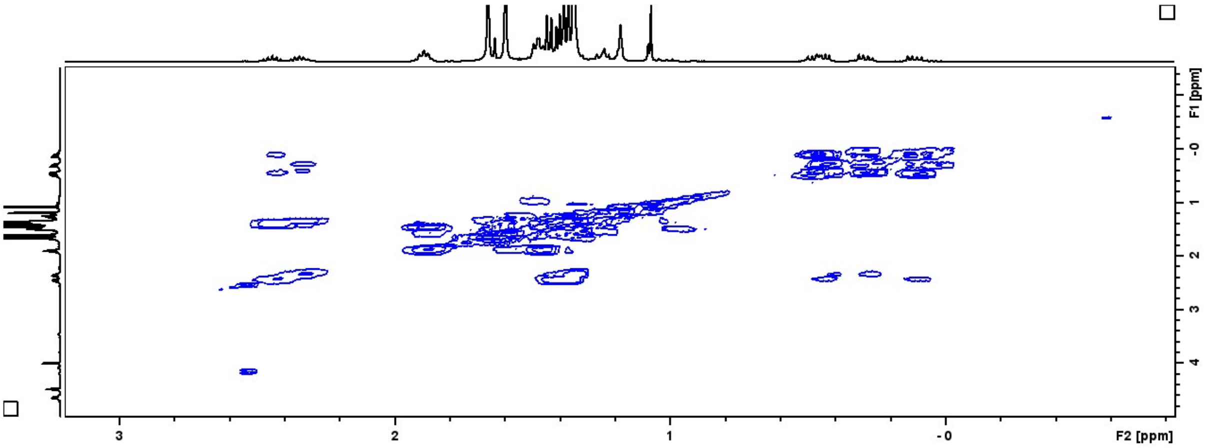

Figure S34: $^1H^1H$-COSY NMR spectrum (expanded) of [Cs(^{t}BuDHP)] + [(TMP)Al(^{i}Bu)$_2$] in C_6D_6

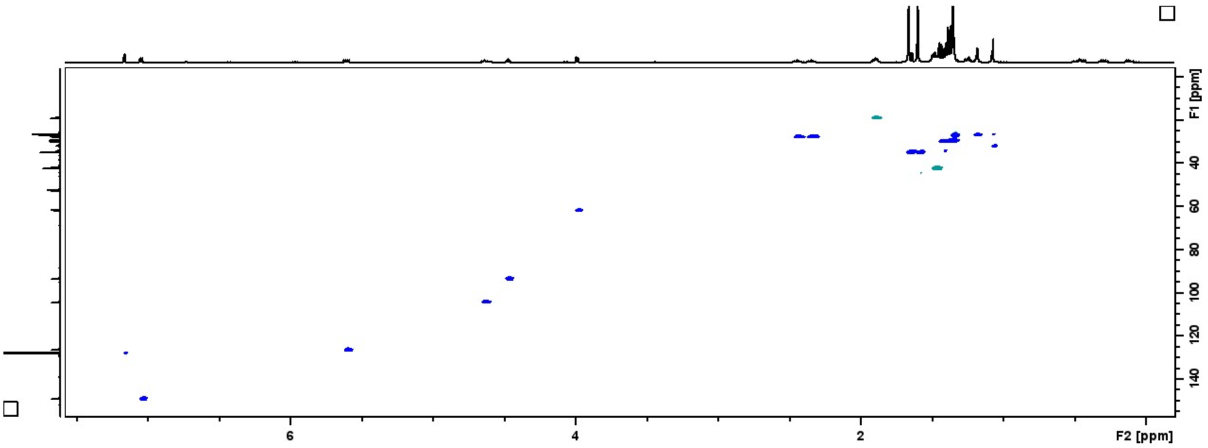

Figure S35: $^1H^{13}C$-HSQC NMR spectrum of [Cs(^{t}BuDHP)] + [(TMP)Al(^{i}Bu)$_2$] in C_6D_6

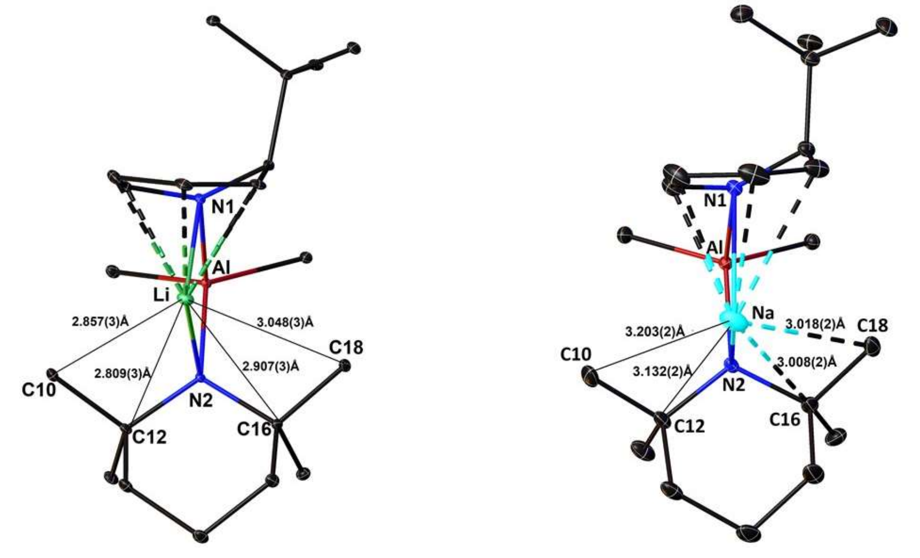

Figure S36: Figure depicting the opposite slipping of compound **1** (left) with respect to compound **2** (right). Li is inclined towards C10 and C12 (trans to the ^{t}Bu group of DHP) unlike C16 and C18 (cis to the ^{t}Bu group of DHP) in case of Na. Carbon atoms from the ^{i}Bu substituent and hydrogen atoms have been omitted for clarity. Thermal ellipsoids are displayed at 30 % probability.

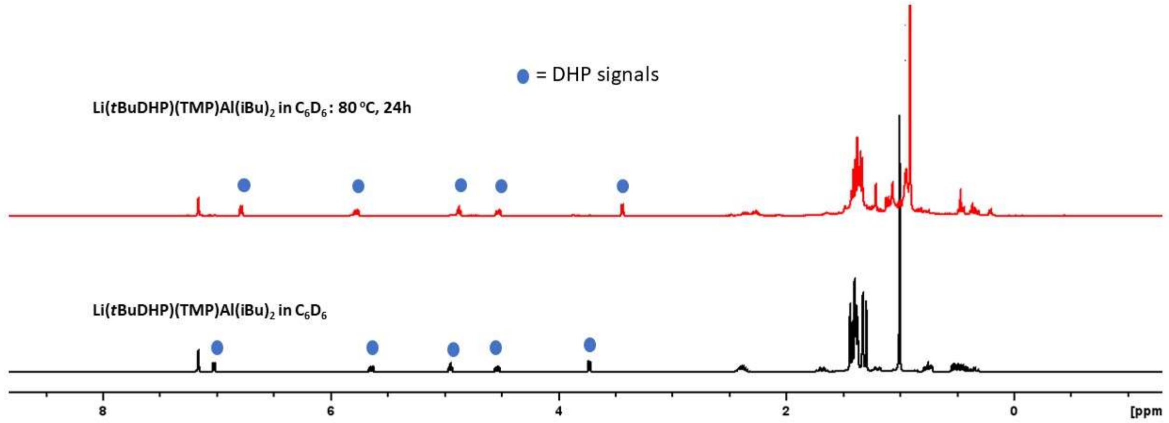

Figure S37: ^{1}H NMR stacking plot depicting thermal stability of Li(*t*BuDHP)(TMP)Al(iBu)$_2$ in C_6D_6

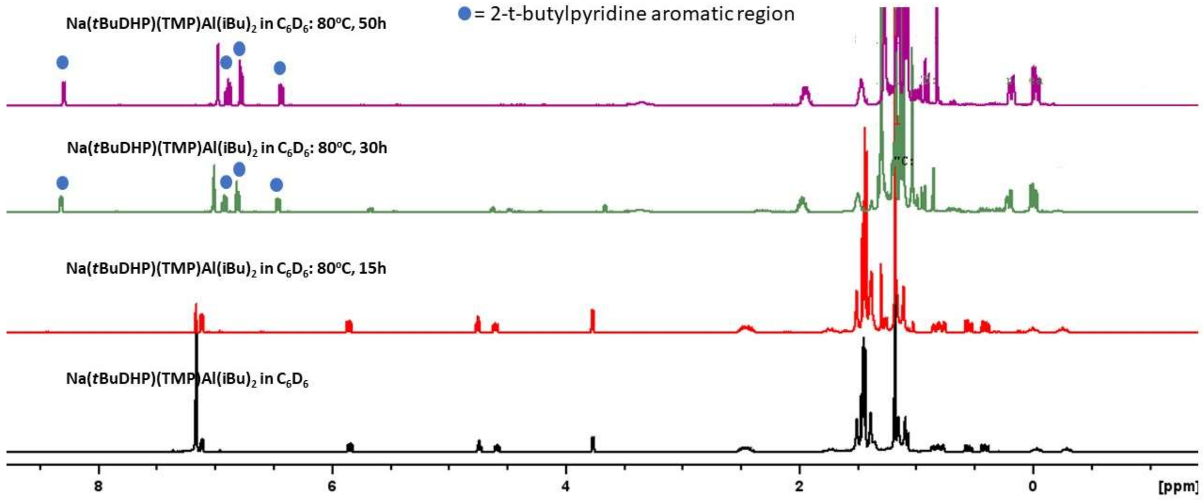

Figure S38: ^{1}H NMR stacking plot depicting thermal decomposition of [Na(*t*BuDHP)(TMP)Al(iBu)$_2$]$_\infty$ in C_6D_6 with generation of 2-t-butylpyridine.

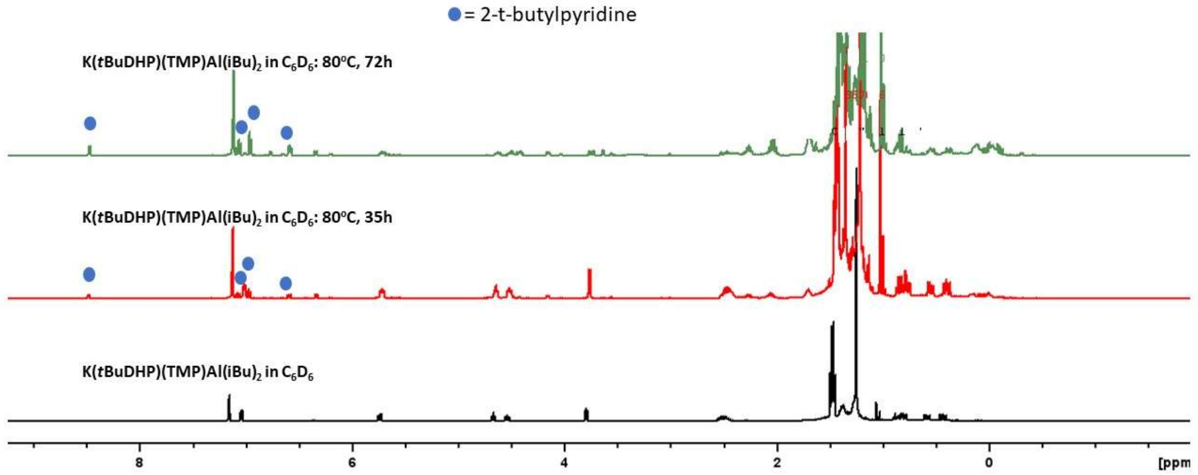

Figure S39: ^{1}H NMR stacking plot depicting thermal decomposition of [K(*t*BuDHP)(TMP)Al(iBu)$_2$]$_\infty$ in C_6D_6 with generation of 2-t-butylpyridine.

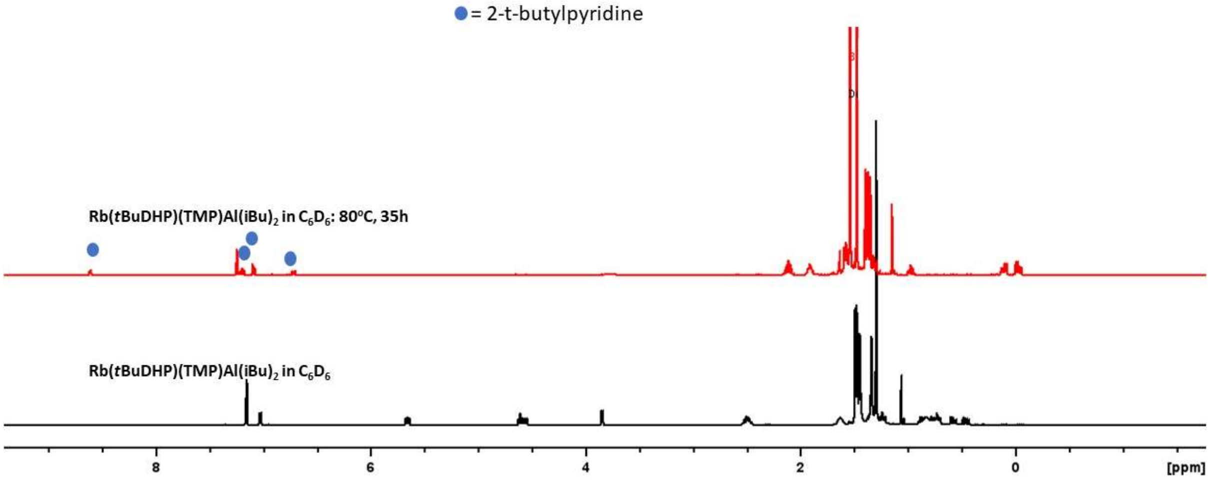

Figure S40: ^{1}H NMR stacking plot depicting thermal decomposition of [Rb(*t*BuDHP)(TMP)Al(iBu)$_2$]$_\infty$ in C_6D_6 with generation of 2-t-butylpyridine.

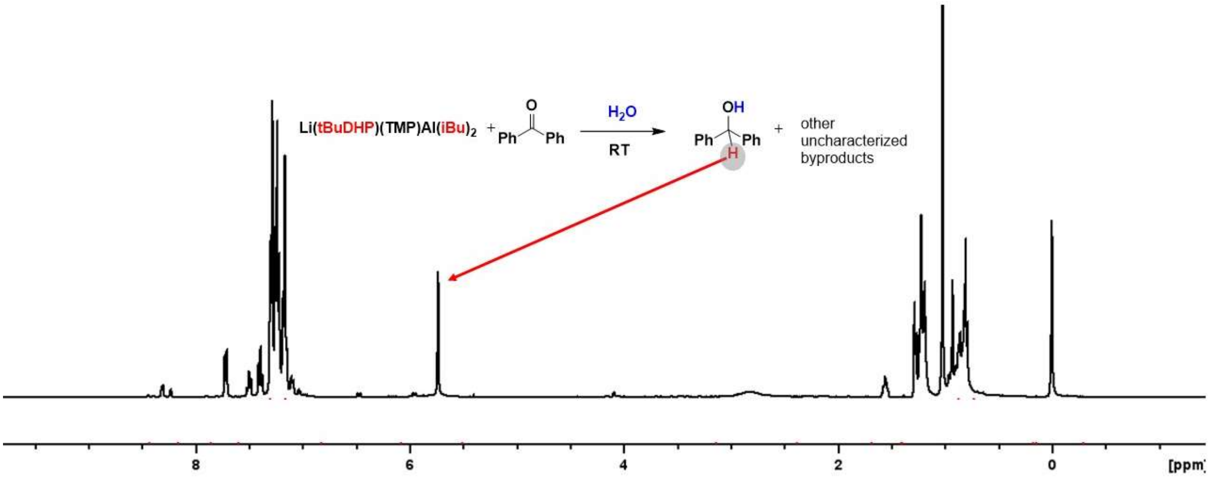

Figure S41: Reaction between Li(*t*BuDHP)(TMP)Al(iBu)$_2$ and benzophenone in a 1:1 ratio in hexane at room temperature followed by quenching with excess amounts of H_2O. ^{1}H NMR spectrum of the aliquot in $CDCl_3$ after evacuating the hexane and other volatiles *in vacuo* depicting the formation of benzhydrol.

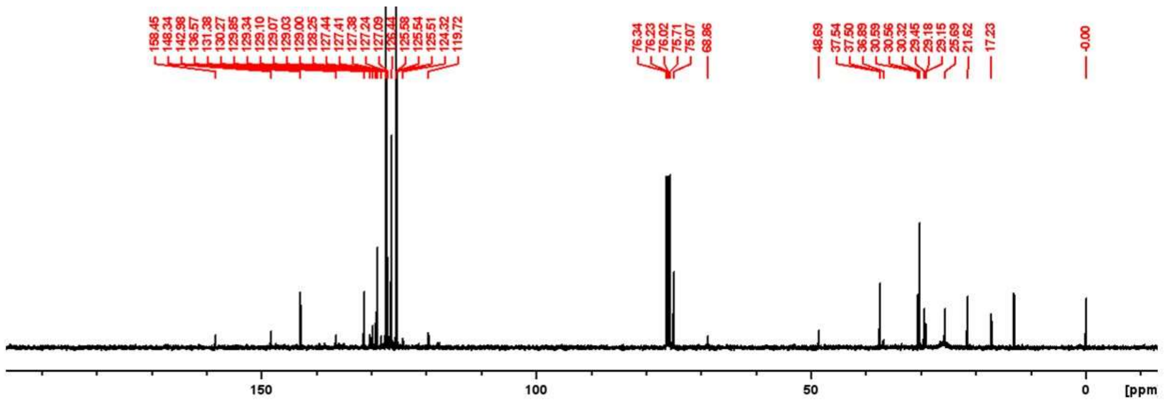

Figure S42: Reaction between Li(*t*BuDHP)(TMP)Al(iBu)$_2$ and benzophenone in a 1:1 ratio in hexane at room temperature followed by quenching with excess amounts of H_2O. ^{13}C NMR spectrum of the aliquot in $CDCl_3$ after evacuating the hexane and other volatiles *in vacuo* depicting the formation of benzhydrol.

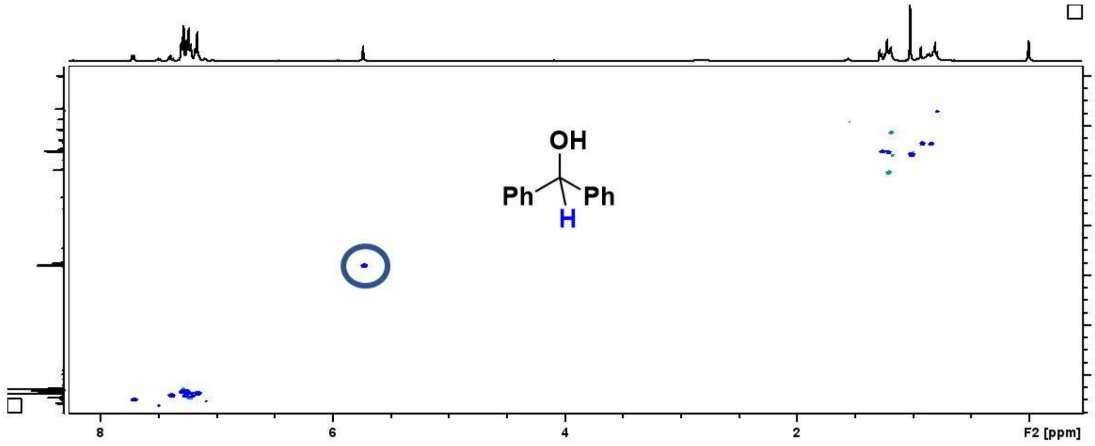

Figure S43: Reaction between Li(*t*BuDHP)(TMP)Al(iBu)$_2$ and benzophenone in a 1:1 ratio in hexane at room temperature followed by quenching with excess amounts of H_2O. ^{1}H^{13}C-HSQC NMR spectrum of the aliquot in $CDCl_3$ after evacuating the hexane and other volatiles *in vacuo* depicting the formation of benzhydrol. The $Ph_2C(\mathit{H})OH$ cross peak has been circled in the figure.

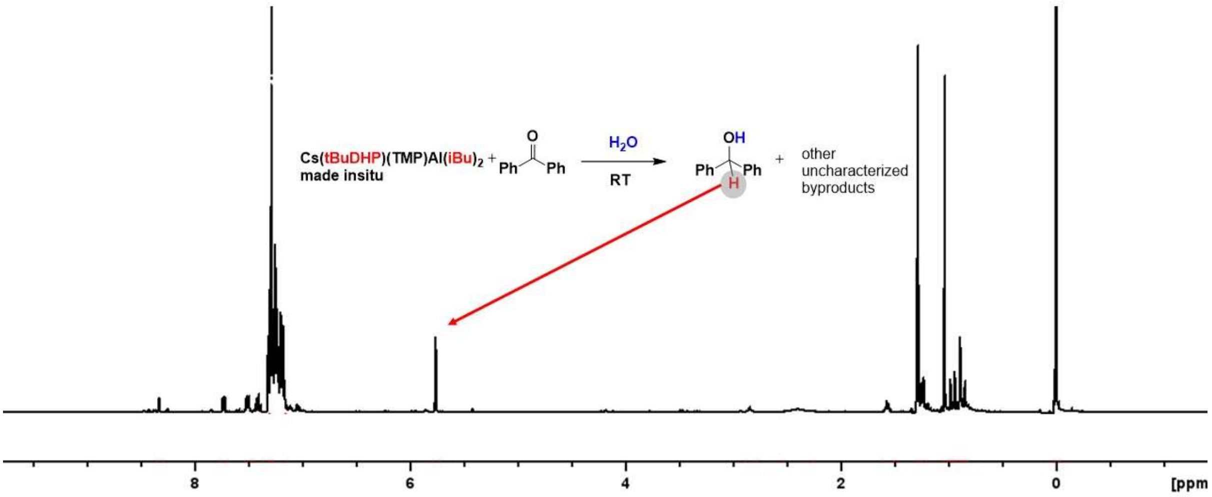

Figure S44: Reaction between [Cs(*t*BuDHP) + (TMP)Al(iBu)$_2$] and benzophenone in a 1:1 ratio in benzene at room temperature followed by quenching with excess amounts of H_2O. ^{1}H NMR spectrum of the aliquot in $CDCl_3$ after evacuating the benzene and other volatiles *in vacuo* depicting the formation of benzhydrol.

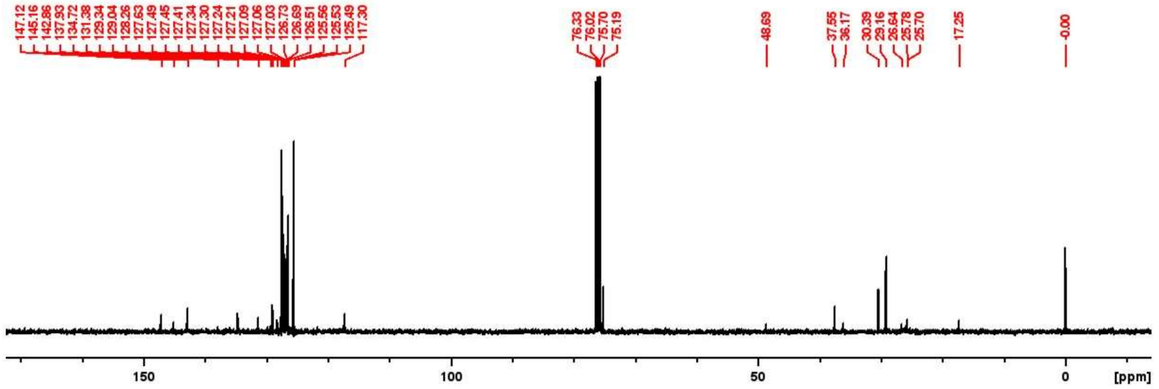

Figure S45: Reaction between [Cs(*t*BuDHP) + (TMP)Al(iBu)$_2$] and benzophenone in a 1:1 ratio in benzene at room temperature followed by quenching with excess amounts of H_2O. ^{13}C NMR spectrum of the aliquot in $CDCl_3$ after evacuating the benzene and other volatiles *in vacuo* depicting the formation of benzhydrol.

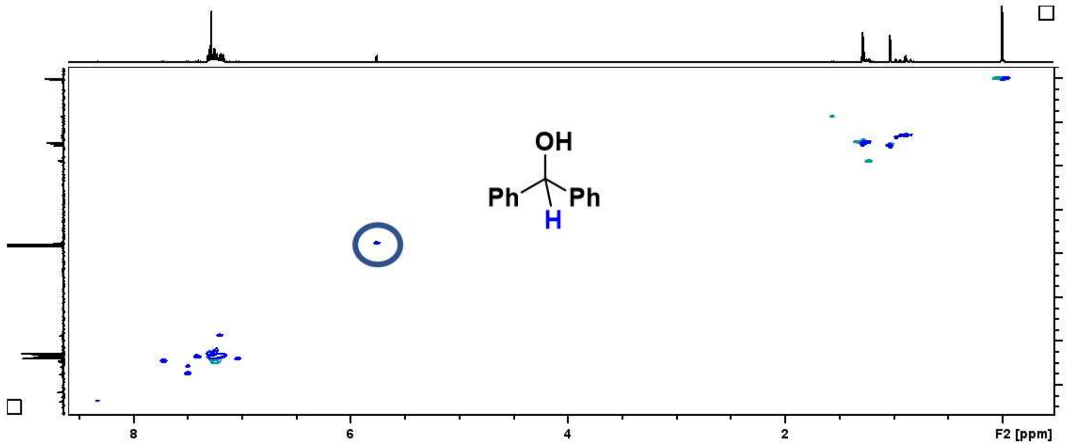

Figure S46: Reaction between [Cs(*t*BuDHP) + (TMP)Al(iBu)$_2$] and benzophenone in a 1:1 ratio in benzene at room temperature followed by quenching with excess amounts of H_2O. $^1H^{13}C$-HSQC NMR spectrum of the aliquot in $CDCl_3$ after evacuating the benzene and other volatiles *in vacuo* depicting the formation of benzhydrol. The $Ph_2C(H)OH$ cross peak has been circled in the figure.

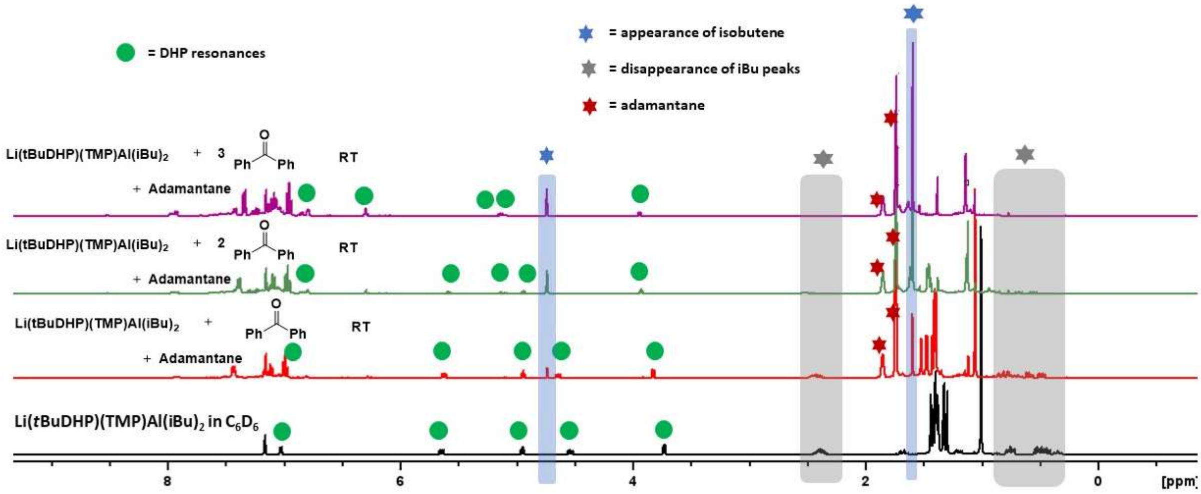

Figure S47: Monitoring surrogate hydride reactivity of compound **1** with benzophenone when added in 1:1, 1:2, and 1:3 equivalents in C_6D_6 at room temperature using ^1H NMR spectroscopy.

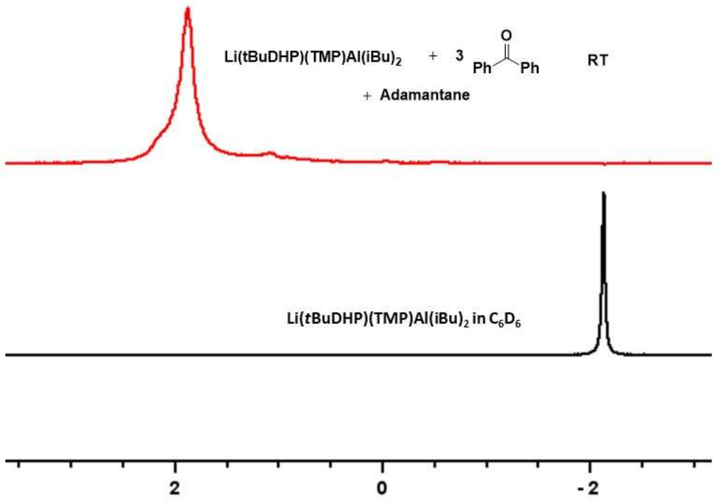

Figure S48: Monitoring the lithium environment before and after adding 3 equivalents of benzophenone to compound **1** using ^{7}Li NMR spectroscopy.

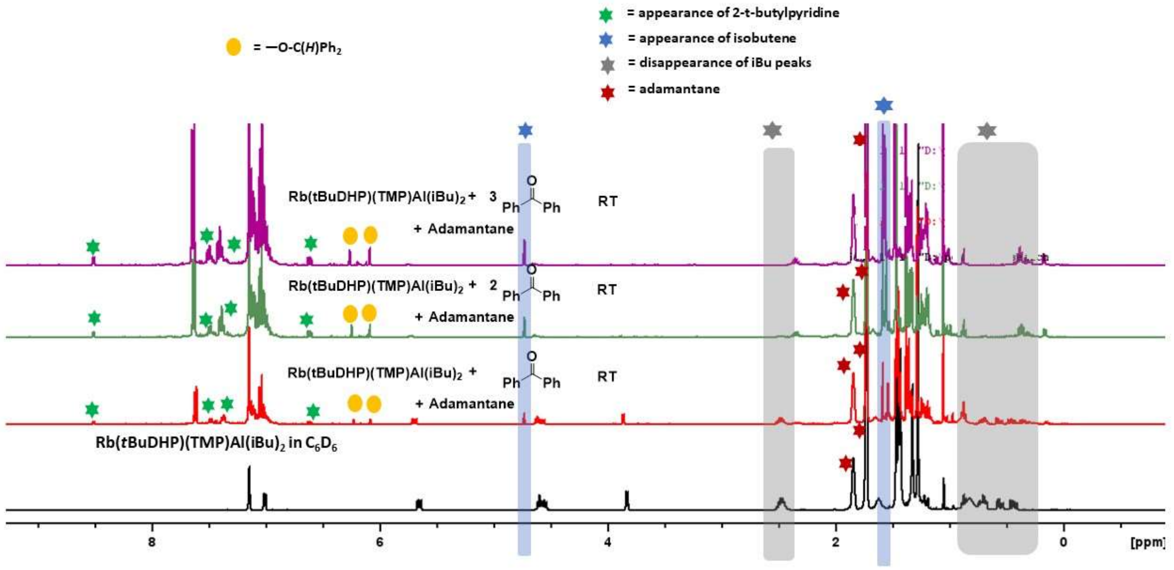

Figure S49: Monitoring surrogate hydride reactivity of compound **4** with benzophenone when added in 1:1, 1:2, and 1:3 equivalents in C_6D_6 at room temperature using ^1H NMR spectroscopy.

Table S1. Selected Crystallographic and Refinement Parameters.

Compound	1	1a	2	2a	3	4
Formula	$C_{26}H_{50}AlLiN_2$	$C_{30}H_{58}AlLiN_2O$	$C_{26}H_{50}AlN_2Na$	$C_{32}H_{66}AlN_4Na$	$C_{26}H_{50}AlKN_2$	$C_{26}H_{50}AlN_2Rb$
Form. Wt.	424.60	496.70	440.65	556.85	456.76	503.13
Space Group	P-1	P-1	$P2_1/c$	$P2_1$	$P2_1/c$	$P2_1/c$
Crystal system	triclinic	triclinic	monoclinic	monoclinic	monoclinic	monoclinic
Temp. (K)	100(2)	123(2)	153(2)	100(2)	100(2)	100(2)
Wavelength (Å)	1.54184	0.71073	0.71073	1.54184	1.54184	1.54184
a (Å)	8.1374(2)	10.6908(7)	10.6567(5)	8.4930(1)	10.6279(1)	10.56024(7)
b (Å)	9.4985(2)	16.3470(11)	11.8845(5)	20.1075(2)	11.7498(1)	11.92130(7)
c (Å)	17.5677(5)	18.8666(13)	21.7676(11)	10.8267(1)	21.7331(2)	21.82898(12)
α (°)	86.496(2)	83.737(6)	90	90	90	90
β (°)	85.490(2)	77.673(6)	96.410(4)	109.968(1)	95.879(1)	96.2551(5)
γ (°)	81.417(2)	77.967(6)	90	90	90	90
Volume (Å^3)	1336.82(6)	3143.294)	2739.6(2)	1737.76(3)	2699.66(4)	2731.73(3)
Z	2	4	4	2	4	4
Measured Reflections	26143	27359	59369	36466	54051	65491
Unique Reflections	4849	13681	6986	6705	5357	5417
R_{int}	0.0552	0.0457	0.0558	0.1149	0.0993	0.0721
Θmax (°)	68.274	27.000	29.000	72.756	72.695	72.748
No. Parameters	318	719	357	424	364	364
S	1.077	0.964	1.056	1.069	1.051	1.075
R [on *F*, obs refs only]	0.0484	0.0602	0.0443	0.0497	0.0449	0.0338
ωR [on F^2, all data]	0.1337	0.1486	0.1180	0.1349	0.1247	0.0906
Largest diff. peak /hole (eÅ^{-3})	0.597/-0.340	0.391/-0.310	0.315/-0.294	0.412/-0.333	0.444/-0.445	0.989/-0.847

References

[1] W. L. F. Armarego, C. Li Lin Chai, Purification of Laboratory Chemicals, Seventh Edition, 2012.

[2] S. D. Robertson, A. R. Kennedy, J. J. Liggat, R. E. Mulvey, *Chem. Commun.* **2015**, *51*, 5452-5455.

[3] S. A. Orr, A. R. Kennedy, J. J. Liggat, R. McLellan, R. E. Mulvey, S. D Robertson, *Dalton Trans.* **2016**, *45*, 6234-6240.

[4] B. Conway, J. García-Álvarez, E. Hevia, A. R. Kennedy, R. E. Mulvey, S. D. Robertson, *Organometallics* **2009**, *28*, 6462-6468.

[5] A. I. Ojeda-Amador, A. J. Martínez-Martínez, A. R. Kennedy, C. T. O'Hara, *Inorg. Chem.* **2016**, *55*, 5719-5728.

[6] 2018, CrysAlisPro Software system, version 1.171.39.46, Rigaku Corporation, Oxford, UK Rigaku Oxford Diffraction Rigaku Oxford Diffraction, **2018**.

[7] O. V. Dolomanov, L. J. Bourhis, R. J. Gildea, J. A. K. Howard, H. Puschmann, *J. Appl. Crystallogr.* **2009**, *42*, 339–341.

[8] G. M. Sheldrick, *Acta Crystallogr. C Struct.* **2015**, *71*, 3–8.

[9] G. M. Sheldrick, *Acta Crystallogr. A* **2008**, *64*, 112–122.

[10] L. J. Farrugia, *J. Appl. Crystallogr.* **2012**, *45*, 849–854.

[11] N. M. O'Boyle, M. Banck, C. A. James, C. Morley, T. Vandermeersch, G. R. Hutchison, *J. Cheminform.* **2011**, *3*, 33.

[12] F. Neese, F. Wennmohs, U. Becker, C. Riplinger, *J. Chem. Phys.* **2020**, *152*, 224108.

[13] M. Kaiser, J. Klett, *Dalton Trans.* **2018**, *47*, 12582-12586.

[14] J. García-Álvarez, E. Hevia, A. R. Kennedy, J. Klett, R. E. Mulvey, *Chem. Commun*. **2007**, 2402-2404.
